# Genome-wide expression profiling of glioblastoma using a large combined cohort

**DOI:** 10.1038/s41598-018-33323-z

**Published:** 2018-10-10

**Authors:** Jing Tang, Dian He, Pingrong Yang, Junquan He, Yang Zhang

**Affiliations:** 1 Innovative Drug Research and Bioinformatics Group, School of Pharmaceutical Sciences and Innovative Drug Research Centre, Chongqing University, Chongqing, 401331 China; 2 Materia Medica Development Group, Institute of Medicinal Chemistry, Lanzhou University School of Pharmacy, Lanzhou, 730000 China; 3 Gansu Institute for Drug Control, Lanzhou, 730070 China

## Abstract

Glioblastomas (GBMs), are the most common intrinsic brain tumors in adults and are almost universally fatal. Despite the progresses made in surgery, chemotherapy, and radiation over the past decades, the prognosis of patients with GBM remained poor and the average survival time of patients suffering from GBM was still short. Discovering robust gene signatures toward better understanding of the complex molecular mechanisms leading to GBM is an important prerequisite to the identification of novel and more effective therapeutic strategies. Herein, a comprehensive study of genome-scale mRNA expression data by combining GBM and normal tissue samples from 48 studies was performed. The 147 robust gene signatures were identified to be significantly differential expression between GBM and normal samples, among which 100 (68%) genes were reported to be closely associated with GBM in previous publications. Moreover, function annotation analysis based on these 147 robust DEGs showed certain deregulated gene expression programs (e.g., cell cycle, immune response and p53 signaling pathway) were associated with GBM development, and PPI network analysis revealed three novel hub genes (RFC4, ZWINT and TYMS) play important role in GBM development. Furthermore, survival analysis based on the TCGA GBM data demonstrated 38 robust DEGs significantly affect the prognosis of GBM in OS (p < 0.05). These findings provided new insights into molecular mechanisms underlying GBM and suggested the 38 robust DEGs could be potential targets for the diagnosis and treatment.

## Introduction

Glioblastomas (GBMs) are the most common and highly aggressive malignant brain tumors^1,2^. Worldwide, in developed countries, an estimated 3~5 GBM cases per 100,000 inhabitants are diagnosed each year^1,3^. 10,000 new cases of malignant GBM are diagnosed each year in the United States^3^. Despite enormous advances in knowledge and therapies over the decades, survival of patients diagnosed with GBM has not significantly improved, only around 5.1% of glioblastoma patients have a 5-year survival rate^4,5^. Therefore, understanding the molecular mechanism of GBMs is an important prerequisite for discovering a novel and effective therapeutic strategy^5–8^.

High-throughput genomic technologies have been widely applied to facilitate to understand the mechanisms involved in the genesis of disease processes^9^. Among which, DNA microarray is recognized as very important and powerful tool for identifying the diversity of functional genes and identifying in-depth characterization of changes in gene expression because it can provide invaluable information on gene transcription by simultaneously measuring expression of thousands of genes within a particular biological sample^10^. For example, Li *et al*.^11^ identified that EZH2 could regulate neuroblastoma cell differentiation via NTRK1 promoter epigenetic modifications using DNA microarrays. And Dmitriy *et al*. discovered listeria species based on the iap gene sequence^12^.

Numerous studies have examined gene expression profiles of individuals with GBM compared with healthy controls and demonstrated that highly proliferation^13,14^, migration^13^, and invasion^15^ nature of GBM cell are key factors hindering effective treatment of gliomas. However, owing highly complexity and intrinsically heterogeneity of GBMs at a molecular level, the specific molecular mechanisms underlying GBM are still poorly understood^16,17^. Recent studies have shown that a robust signature comprising of genes can provide essential basis to study molecular mechanisms that underpin the process of disease^18^. It is reported that a robust signature critically depended on the sample sizes studied^19^, and even need thousands of samples. However, the number of normal control samples in public gene expression databases are disproportionally small compared to tumor samples in a variety of datasets^20^. In other words, the number of normal samples is inadequate for directly identifying the robust differential expression genes associated with GBM.

Herein, the most comprehensive set of genome-scale mRNA expression data was constructed by combining GBM and normal samples from multiple studies. In total, thousands of samples were analyzed to compile the accurate and robust relevant genes towards insight into the molecular mechanisms. In particular, a list of genes with well robustness significantly different between GBM and normal tissue samples was firstly identified. Secondly, functional analysis base on these robust gene sets was performed and certain deregulated gene expression programs (e.g. cell cycle, immune response, p53 signaling pathway) are identified in glioblastoma process. Moreover, enrichment analysis of transcription factors and targeted miRNAs revealed three novel hub genes including RFC4, ZWINT, and TYMS and three transcriptional factors TATA, E2F4DP1 and HFH4, and two microRNA hsa-mir-519E and hsa-mir-527 driving GBM tumorgenicity. Furthermore, survival analysis was applied for evaluating the prognostic performance of these robust differential expression genes using the clinical information of TCGA GBM data. In sum, the identified robust genes may facilitate the understanding of glioblastoma’s etiology and the discovery of novel hub genes, transcriptional factors and two microRNA driving GBM tumorgenicity would have therapeutic implications.

## Materials and Methods

### Data collection and pre-processing

Genome-wide expression data sets were collected based on Human Genome U133 Plus 2.0 Array from Affymetrix GeneChip. In particularly, all raw CEL files of analyzed samples were directly downloaded from two well-known Gene Expression Omnibus (GEO) and ArrayExpress (AE) databases. Annotations information of each sample studied was carefully inspected, including GSM files from GEO database and sdrf files from AE database. All CEL files then were processed using single-channel array normalization (SCAN) method by SCAN.UPC package^21^ with default option in R software. In addition, version 17.0 of BrainArray was used for addressing expression of the same gene with several probes. For gene expression of duplicated samples and only one sample was retained. The final data matrix consisted of expression values for 22215 probes sets and 1588 samples. All detailed descriptions could be found in Lee’s pioneer study^20^.

### Statistical modelling for robust gene signature (RGS) between GBMs and normal samples

Gene expression matrix after combining all studies was furtherly analyzed using bioinformatic methods. Firstly, computing test statistics for expression value of each gene using *mt*.*teststat* function by multtest package of the R statistical computing environment. Secondly, p-value of each gene set were computed using one-sided tests. Then, the resulting p-value for the up and downregulated genes were further adjusted for multiple testing using Benjamini and Hochberg method (BH). Moreover, to identify robust DEGs between GBM and normal controls, the samples size 722 (361 samples each groups)^22^ were randomly selected from a data set of 1,588 samples, and a gene set was prepared by selecting top 500 genes with the lowest p-value from t test analysis. The random sampling was performed 200 times. Secondly, an overlap between two gene-sets was computed for each pair of 200 gene sets. Overlap is the fraction of shared features that appear on both two lists of markers which determined the robustness of the identified markers by measure the similarity of two lists of identified markers^23^. All procedures aimed to identify robust gene signature (RGS) between GBM and normal control samples. Genes repeatedly selected during random sampling are defined as robust^22,24^.

### Hierarchical clustering based on the robust gene signature (RGS)

To determine the specificity of RGS between GBM and normal samples, unsupervised hierarchical clustering analysis^25^ (HCA) was utilized for clustering distinct sample groups. The GBMs and normal samples were clustered by HCA based on the manhattan distance, and the ctc packages in R was furtherly used for converting hclust objects to newick format file. Then, the resulting output was used by the version 3 of Interactive Tree Of Life (iTOL) software to generate the associated heatmap and clustering dendrogram^26^.

### Functional category enrichment analysis

In order to explore biological functions of these differential expression genes, gene set enrichment analysis^27^ (GSEA) was performed based on the 1% most up- and downregulated genes between GBMs and normal samples. GSEA is a computational method that measures whether a known gene set shows significant differences between different biological conditions. Particularly, gene ontology term enrichment analysis was first conducted based the 160 DEGs, which including enrichment for GO ‘Biological Process’, ‘Molecular Function’ and ‘Cellular Component’ terms. Secondly, KEGG pathways enrichment analysis based these genes were also implemented. To investigate the top enriched biological functions and pathways of up and down-regulated genes. One thousand random permutations were performed for each analysis and the threshold of false discovery rate (FDR) was set at 0.05 to allow for investigative discovery.

### Transcription factor and target miRNAs enrichment analysis

GSEA based on DEGs was also carried out to elucidate the significant enriched transcription factor (TF) and miRNAs. One thousand random permutations were performed for each analysis. In addition, TFmiR^28^ was applied for performing integrated analysis of transcription factors (TFs), microRNAs (miRNAs) and genes.

### Construction of gene/protein interaction network and analysis

The Search Tool for the Retrieval of Interacting Genes (STRING) database^29^ has been widely used for exploring protein-protein interactions (PPI). Therefore, the PPI network of DEGs between GBMs and normal samples was constructed and visualized using the STRING online tool, which only included interactions with combined score ≥ 0.4^29,30^. Secondly, the property of PPI network was analyzed using the NetworkAnalyzer module in Cytoscape v3.6.1 software^31^, which could be useful in visualizing biological networks and integrating PPI data. The nodes of PPI network indicated genes and degree suggested the number of interactions of the gene with other genes. In PPI network established, these genes with large degrees (connectivity degree > 5) and high betweenness centrality^32^ were selected and regarded as the hub genes.

## Results and Discussion

### A large-scale GBMs and normal tissues samples is completely collected in this work

**Figure 1:**
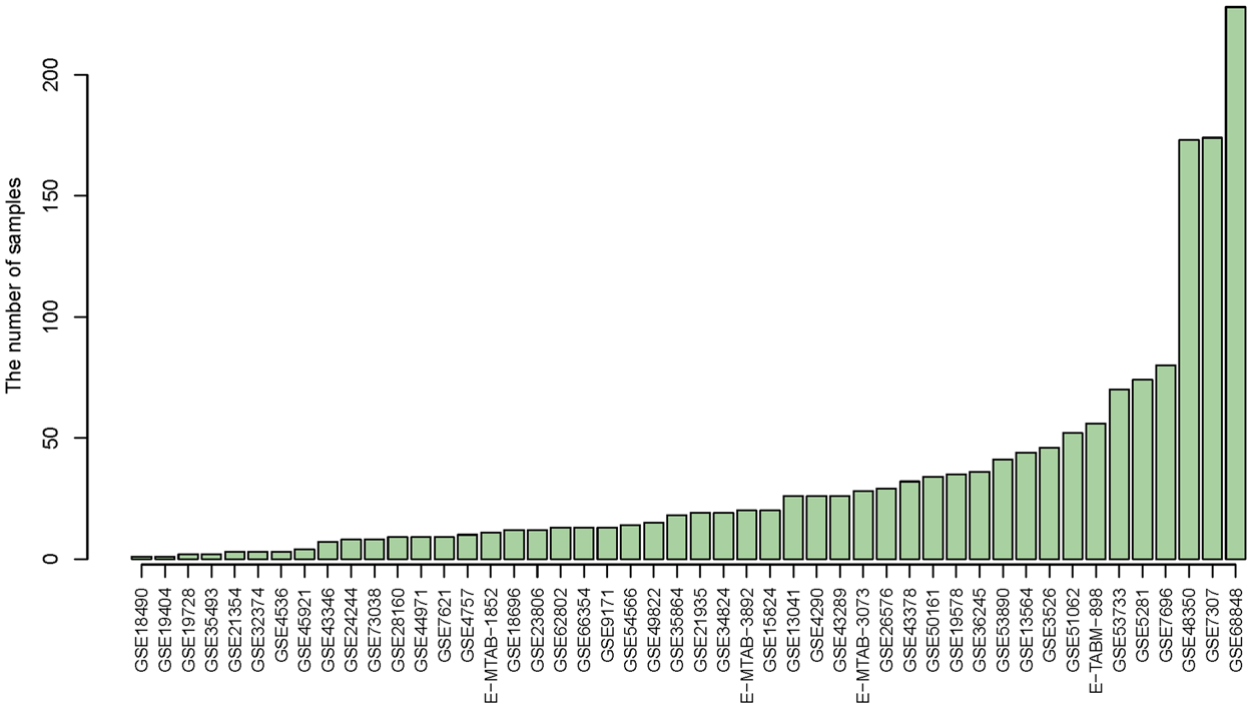
Statistics of datasets studied in this work. Expression profiles of all analyzed samples were collected by Gene Expression Omnibus (GEO) and ArrayExpress (AE) databases. E-MTAB- indicates the AE source; GSE indicates the GEO source. Datasets were ascending ordered by their total number of samples.

Thousands of samples were needed for generating robust differential expression genes associated with disease, which could contribute to understand molecular pathologies and mechanisms of disease^33^. Normal tissues samples can be widely used for cancer-associated studies by providing in invaluable clue for abnormal gene expression patterns in cancer compared to normal^20^. However, the number of normal sample were often small, while the number of cancer samples are relatively large. The unbalance of sample size of distinct groups may limit the study of the disease. Thus, we systematically searched two databases GEO and AE containing publicly-available microarray data sets to obtain the most complete datasets of GBMs and normal tissues samples. The overall numbers of GBMs and normal tissues samples integrating two well-known GEO and ArrayExpress databases was illustrated in Fig. 1. In sum, we collected a total of 48 expression profiling studies, including 723 samples of GBM**s** and 865 samples of normal controls. Among which, the majority of studies either contain GBMs or normal tissue samples separately, e.g. GSE7307 only contained 174 GBMs samples, and GSE68848 only consisted of 228 normal samples.

### Sample size consideration

Statistical power analysis was performed for demonstrating the statistical power of this study. As known, sample size that is too small could reduce the power of a study and increases the probability of error, which can render the study meaningless^34^. Thus, in transcriptomic study, statistical power analysis was typically used in estimating sufficient sample sizes to achieve adequate power^35,36^. For the studied dataset, it has over 90% power to detect differential expression genes at an overall significance level of 0.01 with Bonferroni’s adjustment. As reported in Mapstone’s pioneer study, a power of 0.9 is well suited for detecting differential genes in transcriptomic study^35^. Therefore, the statistical power analysis suggested that the studied dataset (sample size) is well suited for identifying the discriminating genes between GBM cases and normal controls.

### Identify robust and reliable change in gene expression based on thousands of samples

Identification of robust and reliable differential expression genes could provide immense help for understanding molecular mechanisms underlying complex disease. To facilitate the identification of differential expression genes between GBMs and normal samples, p-values were estimated to identify gene sets that were differential expressed between GBMs and control samples. After multiple test correction, the 160 DEGs were selected using the 1% most up and downregulated genes at a false discovery rate of 0.001. The top 10 most significantly up- or down-regulated DEGs were provided in Table 1. Based on the analysis of robust genes above description, a median overlap value was obtained (greater than 0.9), which suggested the identified DEGs are likely to be well robust. In tol, 678 robust genes were repeatedly selected during random sampling. Among of these 678 genes, 147 genes were common identified in the 160 gene-set (1% most up- and downregulated genes between GBMs and normal samples). Namely, 147 robust gene signatures were identified. The relationship between these 147 robust DEGs with GBM were investigated using manually searching PubMed database. We found that 68% (100 of the 147 unique genes have known connections to GBM (Supplementary Table S1). Among 100 robust DEGs associated with GBM, 80 genes were identified to were differential expression in GBM samples, which included 60 were overexpressed or upregulated in GBM samples,12 were downregulated in GBM samples and 8 were differential expression in GBM without the upregulated or downregulated information. The relationship between the top 10 most significant genes and GBMs was listed in Table 2. These genes have been reported to be associated with the survival, growth, invasive and proliferation characteristics of GBMs cells, for example, suppressing of TMEM45A expression in glioma cells remarkably suppressed cell migration and cell invasion, and GJB6, also known as Cx30 has the potential to influence growth, proliferation and migration of glioma cells. Moreover, downregulated two neural subtype expressed markers were identified such as GABRA1 (Gamma-Amino Butyric Acid A Receptor, Alpha1) and SLC12A5 (solute carrier family 12 member 5)^37^. In addition, some genes have not been reported in GBMs-associated studies such as the down-regulated gene TMEM130 with the lowest P-value (P = 2.51E-286). However, recent studies have shown that overexpression of transmembrane protein could increase migration capacity toward glioblastoma cells such as TMEM18^38^. Thus, gene TMEM130 could be associated with GBM and need further validation in the future.

**Table 1:** The top 10 most significantly up- or down-regulated DEGs between GBM and normal samples.

Gene symbol	Gene description	Fold Change
**(1) Upregulated in GBMs**		
COL3A1	collagen, type III, alpha 1	5.628
TOP2A	topoisomerase (DNA) II alpha 170 kDa	8.713
CRISPLD1	cysteine-rich secretory protein LCCL domain containing 1	4.072
RRM2	ribonucleotide reductase M2	9.195
COL1A2	collagen, type I, alpha 2	3.750
FCGBP	Fc fragment of IgG binding protein	4.004
CDCA7L	cell division cycle associated 7-like	4.539
SMC4	structural maintenance of chromosomes 4	5.165
TMEM45A	transmembrane protein 45 A	4.857
PTX3	pentraxin 3, long	8.298
**(2) Downregulated in GBMs**		
MAL2	mal, T-cell differentiation protein 2 (gene/pseudogene)	0.252
GJB6	gap junction protein, beta 6, 30 kDa	0.285
NEFM	neurofilament, medium polypeptide	0.419
SYNPR	synaptoporin	0.407
TMEM130	transmembrane protein 130	0.334
GABRA1	gamma-aminobutyric acid (GABA) A receptor, alpha 1	0.335
RBFOX1	RNA binding protein, fox-1 homolog (C. elegans) 1	0.399
SLC12A5	solute carrier family 12 (potassium/chloride transporter), member 5	0.440
NEFH	neurofilament, heavy polypeptide	0.406
AK5	adenylate kinase 5	0.398

A final set of linear models were used to identify genes that were differential expressed between glioblastoma and control samples. After multiple test correction we identified 1% most up and downregulated genes at a false discovery rate of 0.001.

**Table 2:** The top 10 most significantly up- or down-regulated DEGs between GBMs and normal samples are associated with the GBMs.

Gene symbol	Descriptions of gene is associated with GBM	UP/Down	Ref.
COL3A1	COL3A1 may be suitable biomarkers for diagnostic or therapeutic strategies for GBM	DN	^56^
TOP2A	Over-expression of TOP2A as a prognostic biomarker in patients with GBM	UP	^57^
CRISPLD1	UN	UN	
RRM2	BRCA1-mediated RRM2 expression protects GBM cells from endogenous replication stress	UP	^58^
COL1A2	COL1A2 is highly expressed genes in GBM spheroids as compared with normal brain	UP	^6^
FCGBP	Primary glioblastomas exhibited higher expression of extracellular response-associated gene FCGBP	UP	^59^
CDCA7L	It has been reported that CDCA7L is correlation to GBM patient survival time	UP	^60^
SMC4	Overexpression of SMC4 activates TGFβ/Smad signaling and promotes aggressive phenotype in GBM cells	UN	^61^
TMEM45A	Suppressing of TMEM45A expression in glioma cells remarkably suppressed cell migration and cell invasion	UN	^62^
PTX3	Knockdown of PTX3 significantly decreases GBM8401 cell migration and invasion	UN	^63^
MAL2	UN	UN	
GJB6	GJB6 (Cx30) has the potential to influence growth, proliferation and migration of GBM cells.	UN	^64^
NEFM	KLF6 inhibits the malignant phenotype of GBM *in vitro* and upregulates neuronal marker NEFM.	UP	^65^
SYNPR	SYNPR is downregulated differently expressed genes (DEGs) in GBM tissue samples.	Down	^66^
TMEM130	UN	UN	
GABRA1	Upregulation of miR-155 in GBM could may downregulate GABRA1 which renders tumor cells unresponsive to GABA signaling.	Down	^67^
RBFOX1	Downregulated RBFOX1 is identified in GBMs compared with normal brain.	Down	^68^
SLC12A5	UN	UN	
NEFH	miR-25 promotes GBMs cell proliferation and invasion by directly targeting NEFL.	UN	^69^
AK5	UN	UN	

UP indicated that the gene was identified as up-regulated in GBMs; Down indicated that the gene was reported as down-regulated. UN suggested the gene has not been reported in current GBM-associated studies.

### Hierarchical clustering analysis of differentially expressed GBMs signature genes

**Figure 2:**
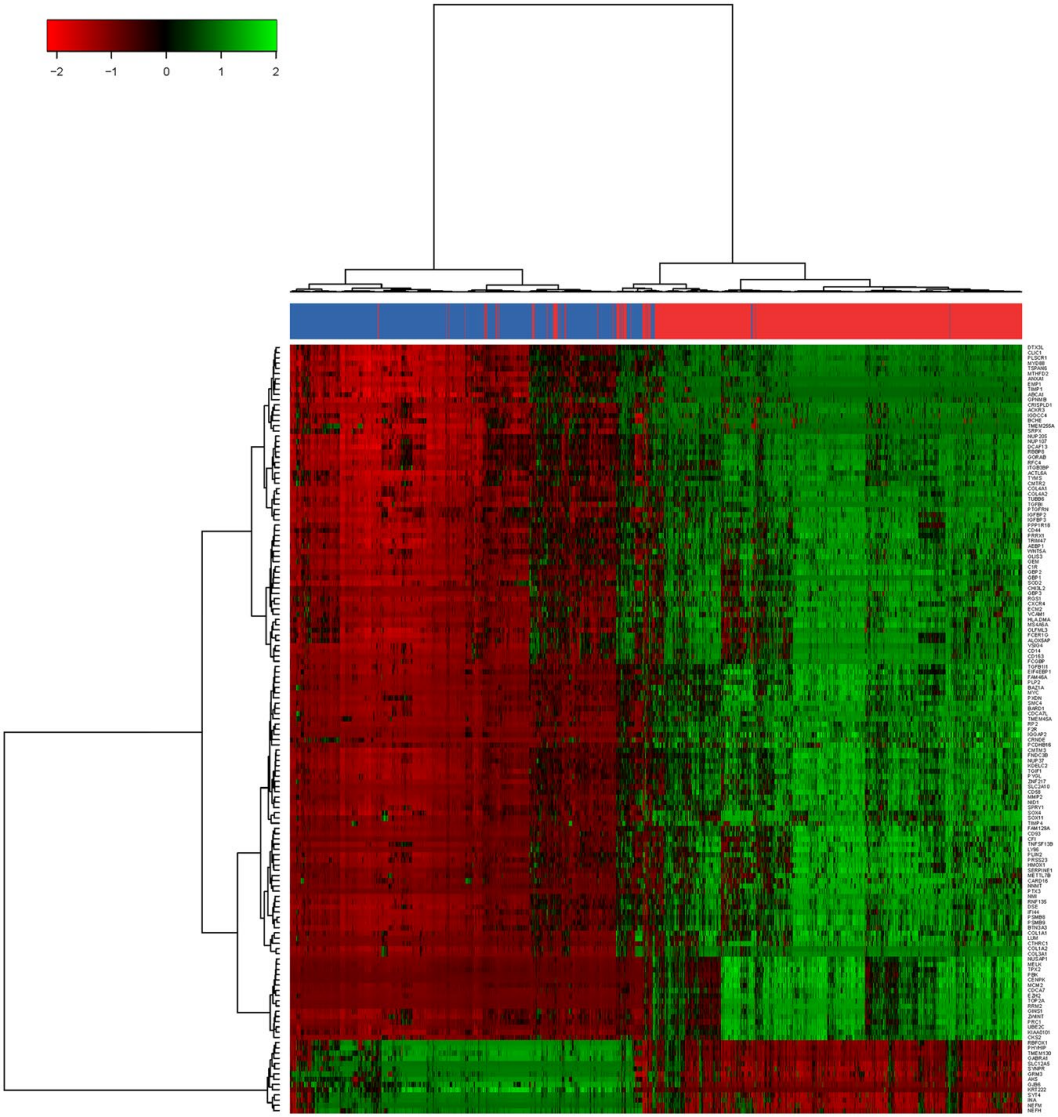
Heatmap of 723 glioblastoma and 865 normal samples based on identified 147 robust differential expression (up and downregulated) genes. The highest expression values of DEGs are displayed in green and the lower gradually fading toward black color. The lowest expression values of DEG are shown in red, higher ones gradually fading toward black color. Glioblastoma samples were highlighted with red; Normal control samples were highlighted with blue.

Unsupervised hierarchical clustering analysis is one of the most powerful methods for the exploratory analysis of gene expression data and was widely used to reflecting distinct gene expression patterns or modules of highly co-expressed genes. Therefore, in this work, hierarchical clustering with ward algorithm^39^ was applied to cluster the expression profile of differentially expressed genes in each sample group based on these 147 robust DEGs including upregulated and downregulated genes in GBMs. As shown in Fig. 2, two subtypes of all samples studied were identified by unsupervised hierarchical clustering. The heatmap demonstrated the most of GBMs and normal samples could be separated based these DEGs.

### Functional analysis of differentially expressed GBMs signature genes

**Figure 3:**
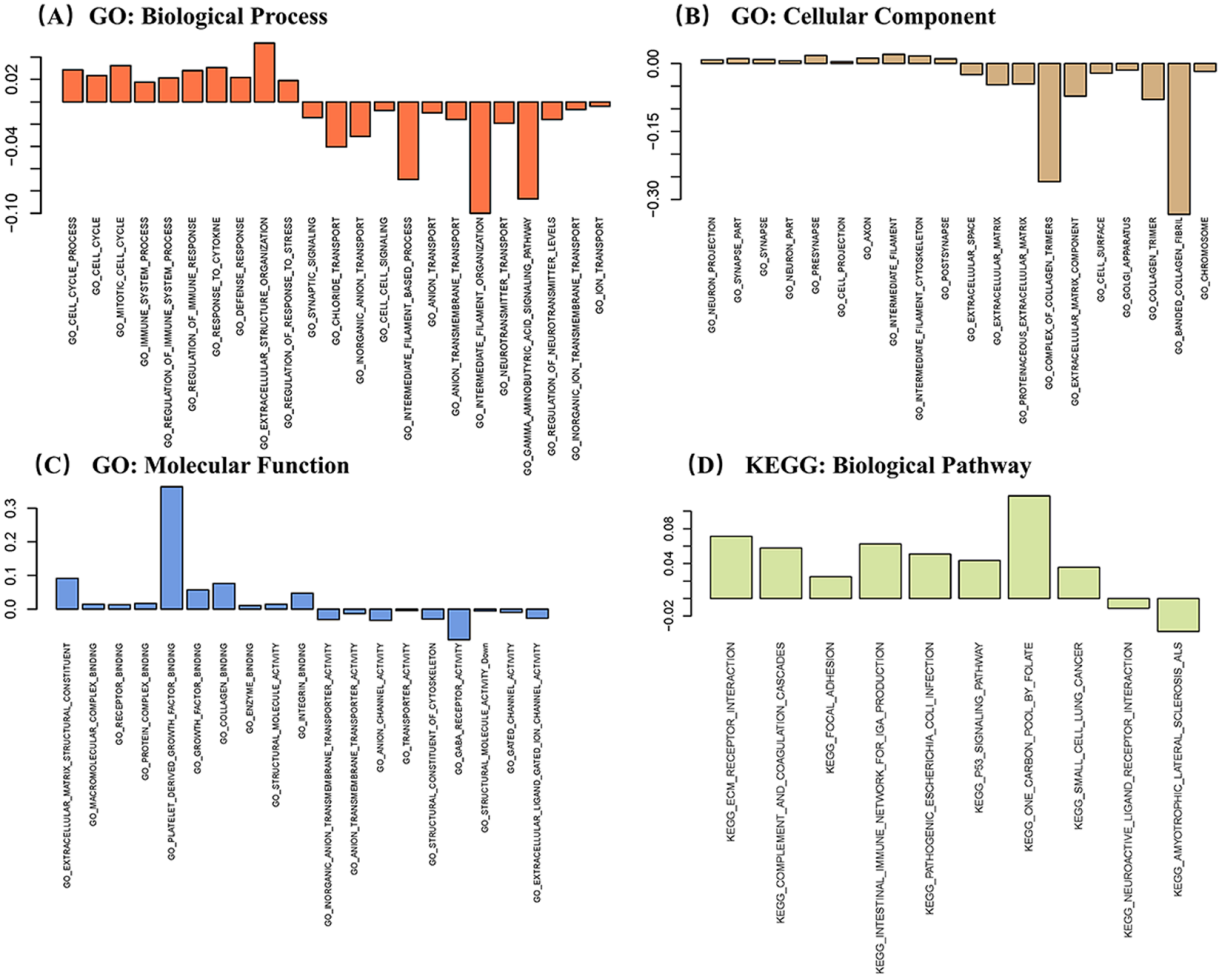
Functional enrichment analysis of gene ontology terms and kegg biological pathway enrichment analysis of DEGs. Gene Ontology covers three domains: cellular component, molecular function and biological process. A-C GO analysis according to biological process, cellular component and molecular function, respectively. (**A**) Enrichment for GO ‘Biological Process’ terms of genes detected. The y-axis displays the fraction relative to all GO Biological Process terms. (**B**) Enrichment for GO ‘Molecular Function’ main terms of genes detected. The y-axis displays the fraction relative to all GO Cellular Component terms. (**C**) Enrichment for GO ‘Molecular Function’ main terms of genes detected. The y-axis displays the fraction relative to all GO Molecular Function terms. The figure shows terms on the x-axis that are significantly enriched (FDR < 0.05). (**D**) Enrichment for kegg ‘Biological Pathway’ terms of genes detected.

Functional analysis is secondary analysis of differential expression genes identified and can collectively provide biological function underlying these genes. Understanding dysregulated biological process and pathway in cancer cells are essential for the development of complex diseases^40^, and can provided immense assistance in the understanding the pathology^41^. Therefore, GSEA was performed to investigate biological function and pathways of genes associated with GBM. As shown in Fig. 3, the BP terms of GO (Fig. 3A) showed that the up-regulated genes were enriched over 50 terms and the top 10 terms were associated with cell cycle and immune response. And the down-regulated genes showed 13 terms enrichment for cell signaling, anion transport, neurotransmitter transport and so on. The CC terms of GO (Fig. 3B) showed that the up-regulated genes were significantly enriched in 32 terms and the top 10 terms were associated with extracellular space, extracellular matrix, complex of collagen trimer, cell surface, golgi apparatus, collagen trimer and banded collagen fibril. And the down-regulated genes showed 31 terms enrichment for neuron projection, cell projection, intermediate filament and synapse, and so on. The MF terms of GO (Fig. 3C) showed that downregulated genes could be associated with transporter activity, anion channel activity, receptor activity and structural molecule activity, whereas the most upregulated genes were associated with protein complex, receptor, growth factor, enzyme binding. In addition, the KEGG pathways analysis based on these DEGs suggested that downregulated genes were significantly enriched in ecm receptor interaction, complement and coagulation cascades, p53 signaling pathway, focal adhesion, immune network and so on. And the down-regulated DEGs showed 2 pathways enrichment for neuroactive ligand receptor interaction and amyotrophic lateral sclerosis als.

### Transcription factor and target miRNAs analysis

Transcription factor (TF) and microRNA (miRNA) are essential for regulating the expression of gene^42^. Differentially expressed TFs in GBM, and their downstream gene targets, may be potential therapeutic biomarkers of GBM. Therefore, we perform the transcription factors enrichment analysis based on these DEGs. As the demonstrated Supplementary Table S2, top 10 TFs based unregulated DEGs and top 10 TFs based on downregulated DEGs are enrichment and listed by GSEA software at FDR < 0.05. The most of TFs reported that the directly associated GBMs. For example, the identified transcription factors FOXD3 could inhibit proliferation, migration, and invasion of GBM cells^42^. Moreover, numerous studies showed that miRNAs played important roles in development of cancer and could be potential targets for therapy^5^. Therefore, to investigate the regulatory mechanisms, we also performed miRNAs enrichment analysis based on these DEGs. As the demonstrated Supplementary Table S3, 16 miRNAs sets are enriched and list by GSEA software at FDR < 0.05. Similarity, the most of miRNAs has reported that the directly associated GBMs. For example, miR-196b was upregulated in GBM compared with normal control samples and associated with cell proliferation^42^.

**Figure 4:**
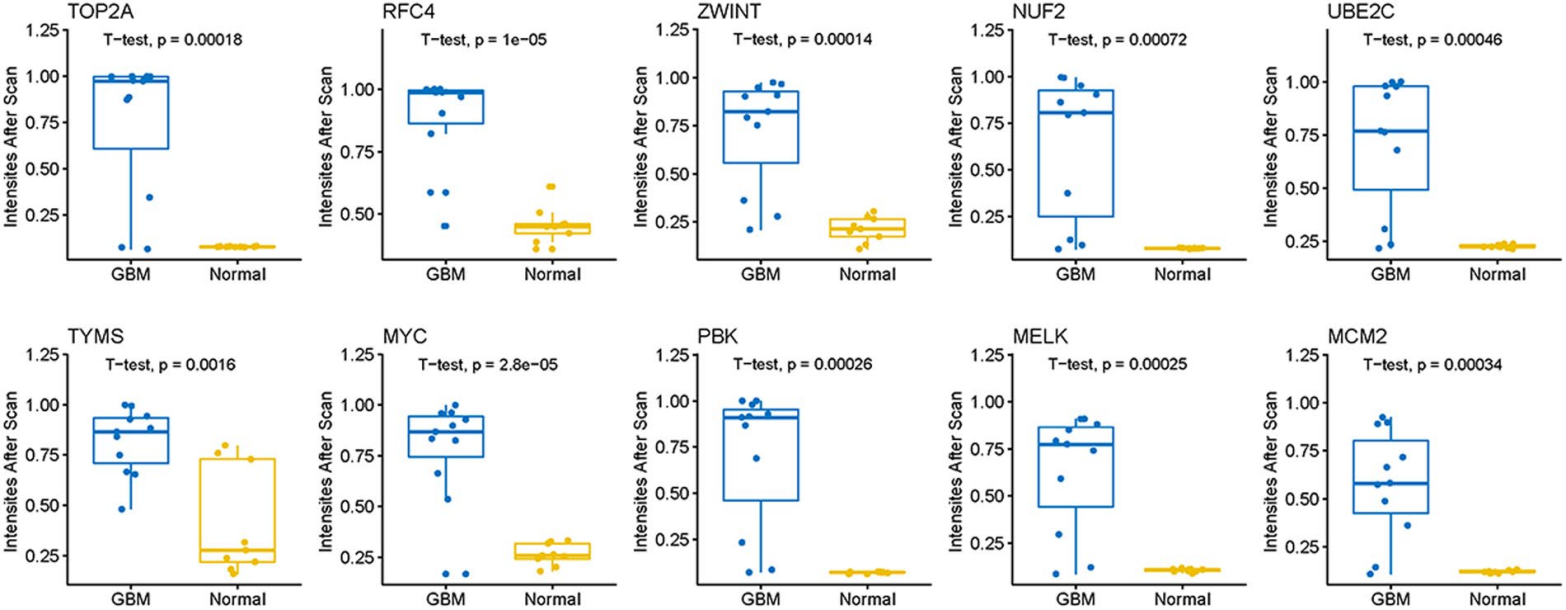
Glioblastoma-specific miRNA/transcription factor co-regulatory networks. The miRNAs are from the enrichment result based on DEGs (top 1% upregulated) at a false discovery rate of 0.05. Green hexagon indicates the transcript factor, the yellow circle represents miRNA, the orange quadrilateral suggests target gene.

In addition, combination analysis of TFs and miRNAs play important roles in understanding the pathogenic mechanisms associated with GBM tumorigenesis^28^. Therefore, we constructed and analyzed co-regulatory network based on enriched TFs and miRNAs using well known web server TFmiR^28^. As the illustrated Fig. 4, a total of 55 regulatory interactions were identified which included 29 nodes (miRNAs and TFs/genes). Among which, 28 interactions were experimentally validated. The over representation analysis of the full interaction network showed 9 targeted miRNAs including hsa-mir-106a, hsa-mir-130a, hsa-mir-196b, hsa-mir-20a, hsa-mir-20b, hsa-mir-29a, hsa-mir-29c, hsa-mir-381 and hsa-mir-19a involvement in cancerogenesis of GBMs.

### PPI network construction

**Figure 5:**
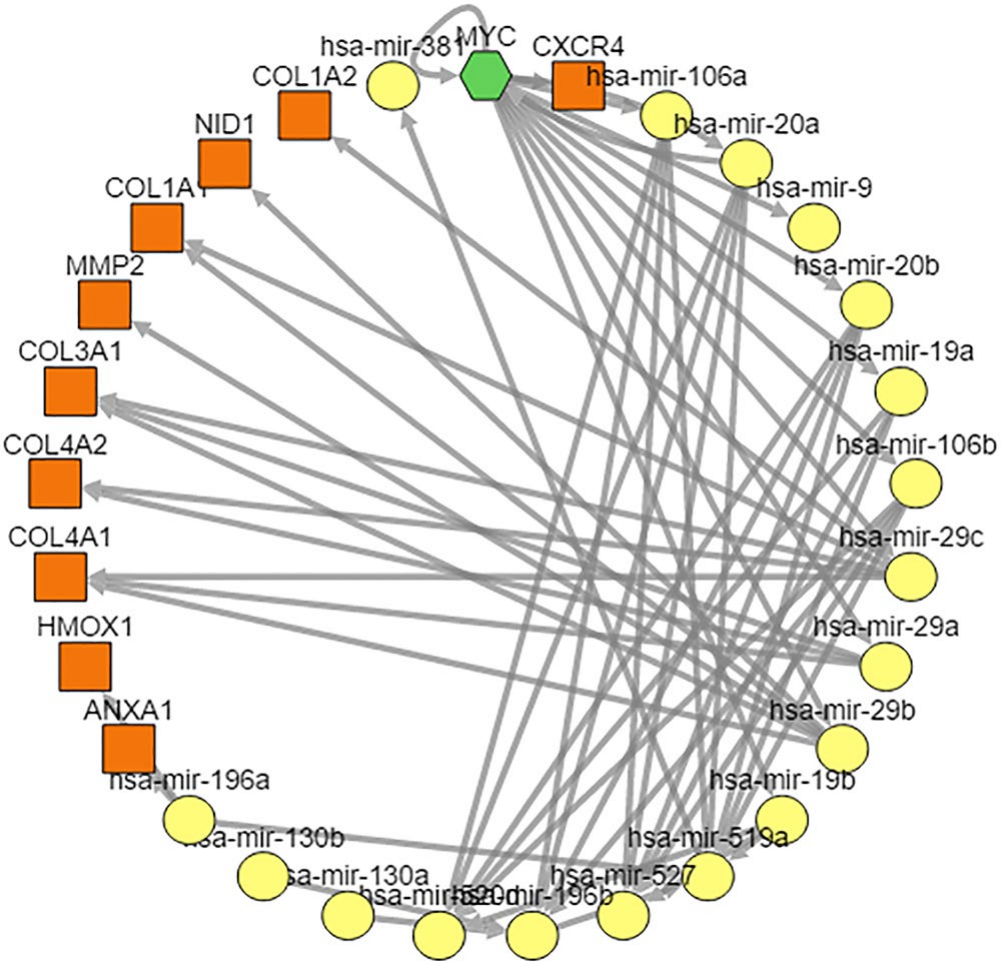
Box plot of intensities after Scan normalization based on top 10 hub genes. Box plot showing median, interquartile range, minimum and maximum intensities with GBMs (blue boxes) compared to those with normal tissue sample (yellow boxes). Corresponding intensities values are displayed as dots. The p-value indicated significant differences between the distinct groups, which is calculated using t-test based on *stat_compare_mean* function in R ggpubr library.

Deciphering the structure of complex network of protein-protein interactions (PPI) can facilitate to understand of molecular mechanism behind the disease^43^. The hub genes of whole PPI network identified play a vital role in this signal transduction network. Therefore, in this work, we preformed PPI network analysis by choosing a well-known high-throughput STRING dataset^44^, which can further promote to select reliable edges of network. Particularly, the PPI network was constructed and visualized based on 1% up and down regulated DEGs, which included 158 nodes and 378 edges. As shown in Supplementary Fig. S1, nodes with high betweenness centrality and large degree (connectivity degree > 5) are selected as hub genes and were displayed in Table 3. Table 3 showed the top 10 crucial hub genes involved in the development of GBMs, which included TOP2A, RFC4, ZWINT, NUF2, UBE2C, TYMS, MYC, PBK, MELK and MCM2. Overall expression values of these hub genes were visualized by boxplot for the E-MTAB-3892 dataset. The obvious gene expression difference between GBM and normal samples could be seen in Fig. 5. Among which, the most of hub genes have been experimentally validated in GBM-associated studies, which reflected the hub genes identified is well reproducibility with previous findings. To be more specific, three hub genes including PBK (role in cell cycle), MELK (stem cell marker), and TOP2A (proliferation marker) have been validated in previous GBM-associated studies^45^. PBK was candidate can be a promising molecular target for GBM treatment^46^. MELK was identified for encoding other ABC transporters as well as Akt3 kinase in developing resistance of GBM to TMZ^47^. TOP2A was the hub protein of whole network, which have been demonstrated to its expression was correlated with aggressive and highly proliferating cancers, which were accordance with Horvath et a’ work. In addition, recent studies have showed that overexpression of MCM2 gene could be highly associated with survival of GBM^48^. Upregulated UBE2C gene was associated with the aggressive progression of GBM^49^. And siRNA-mediated knockdown against NUF2 may be a potential therapeutic method for treatment of GBM^50^.

**Table 3:** The top 10 hub genes with a connectivity degree >5 were selected and listed.

Hub gene	Gene description	Degree	Betweenness centrality
TOP2A	topoisomerase (DNA) II alpha 170 kDa	30	0.2268
RFC4	replication factor C (activator 1) 4, 37 kDa	27	0.0491
ZWINT	ZW10 interactor	22	0.0069
NUF2	NUF2, NDC80 kinetochore complex component, homolog (S. cerevisiae)	22	0.0263
UBE2C	ubiquitin-conjugating enzyme E2C	22	0.0559
TYMS	thymidylate synthetase	21	0.0508
MYC	v-myc myelocytomatosis viral oncogene homolog (avian)	21	0.4453
PBK	PDZ binding kinase	21	0.0415
MELK	maternal embryonic leucine zipper kinase	20	0.0016
MCM2	minichromosome maintenance complex component 2	19	0.0010

Given that the majority of the networks were scale-free, hub genes with a connectivity degree >5 were selected, as described previously. The connectivity degree represents the number of lines linked to a given node, and nodes with a high connectivity degree (≥5) are defined as hub genes that possess important biological functions. All the properties were computed based on these 1% most up and downregulated genes by NetworkAnalyzer module in Cytoscape software.

However, the association between three hub genes including RFC4, ZWINT and TYMS expression and GBMs has not been reported. A recent study by Jiang *et al*.^51^ showed that miR-127-3p and its targeted gene SKI could be promising targets for GBM therapy. The present study revealed that hub gene ZWINT also is a target of miR-127-3p, which has functional annotation related to cell cycle, cell division and nuclear division. Therefore, the gene may be a key regulator in GBM development.

### Survival analysis

**Figure 6:**
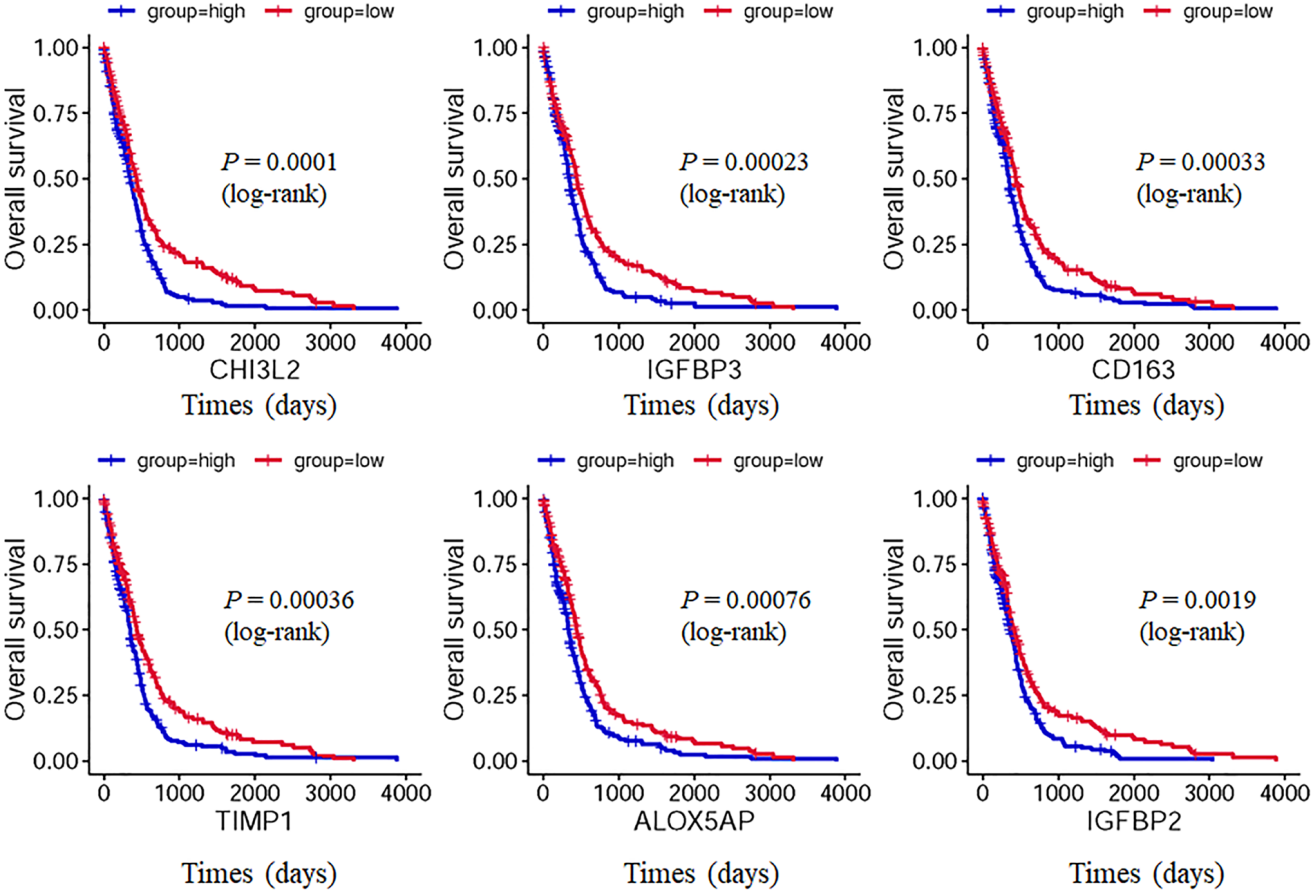
Univariate survival analysis in GBM stratified by robust differential expression gene expression based on the TCGA data as determined by Kaplan-Meier estimates. 521 GBM cases with full data of both clinical and gene expression were collected from the TCGA database. The expression values of these genes were classified as either high (expression value ≥ median) or low (expression value < median). Kaplan-Meier estimates (log-rank test) were made and found 38 genes expression were significantly affect the prognosis of GBM in OS (p < 0.05) (only listed top six genes). More relevant genes were shown in Supplementary Fig. S2.

Investigating the clinical significance (e.g. prognosis) of gene expression in GBM is crucial important for diagnosis and molecular target therapy of GBM^52^. As known, survival analysis was widely applied method to evaluate the prognostic performance of new biomarkers using the clinical data of oncological patients^53^. The Cancer Genome Atlas (TCGA) project is one of the largest available resources that accumulates genomic, transcriptomic and methylomic data for several types of cancer^54^. The TCGA provide a useful source of information for identification of prognostic markers^55^. Therefore, to investigate the oncogenic role of the robust differential expression genes in GBM progression, survival analysis by Kaplan-Meier estimates stratified by their expression was made based on the data of 521 GBM cases provided by TCGA. The expression values of these genes were classified as either high (expression value ≥ median) or low (expression value < median). As the demonstrated Supplementary Fig. S2, we found 38 robust DEGs were significantly related to the prognosis of GBM (OS, P < 0.05) based on Kaplan-Meier estimates (log-rank test). Figure 6 only listed top six significant genes. Thus, these genes are possible candidate genes for diagnosis and molecular target therapy of GBM. Moreover, these 38 robust DEGs significantly associated with overall survival (p < 0.05) in TCGA were retained for further analysis. Cox multivariate model was carried out with function “coxph” in the R package “survival” to develop the risk score model. As demonstrated Table 4, 20 robust DEGs were identified to be with positive coefficients, which could indicate their high expression positively correlated the risk score value, thus, these genes might be tumor genes. While 18 robust DEGs were identified to be with negative coefficients, which could indicate their high expression negatively correlated the risk score value, thus, these genes might be tumor suppressor genes. The performance of the risk score was evaluated by dividing the GBM samples in the TCGA into two subgroups, high-risk and low-risk, using the median risk score as a cutoff (2.992). As illustrated Supplementary Fig. S3, the survival time of the low risk score group is significantly longer than the high-risk score group.

**Table 4:** Parameters of gene symbol, Hazard ratio, p values, coefficients and 95% confidence interval of 38 genes according to Cox multivariate regression.

Gene symbol	Hazard ratio	*p-*value	Coefficients	*95%* confidence interval
ABCA1	1.064	0.571	0.062	0.858~1.319
AEBP1	1.144	0.025	0.134	1.017~1.287
ALOX5AP	1.023	0.775	0.023	0.875~1.195
CD14	1.41	0.006	0.343	1.101~1.805
CD163	1.027	0.747	0.027	0.872~1.21
CD44	1.13	0.19	0.122	0.941~1.356
CFI	0.989	0.864	−0.011	0.872~1.122
CHI3L2	0.986	0.775	−0.014	0.896~1.086
CLIC1	0.972	0.836	−0.029	0.742~1.274
COL1A1	0.991	0.914	−0.009	0.842~1.166
COL1A2	0.846	0.024	−0.167	0.732~0.979
CXCR4	1.119	0.122	0.112	0.97~1.29
ECM2	0.982	0.769	−0.018	0.872~1.106
FCER1G	0.908	0.573	−0.096	0.65~1.268
FNDC3B	1.07	0.579	0.068	0.843~1.358
GPNMB	0.991	0.85	−0.009	0.901~1.09
HLA.DMA	0.705	0.002	−0.349	0.568~0.876
HMOX1	0.891	0.081	−0.115	0.783~1.014
IFI44	1.052	0.514	0.05	0.904~1.224
IGFBP2	1.1	0.073	0.095	0.991~1.22
IGFBP3	1.044	0.368	0.043	0.951~1.147
LY96	1.234	0.007	0.21	1.06~1.437
MMP2	0.988	0.863	−0.012	0.859~1.136
MTHFD2	0.892	0.245	−0.114	0.736~1.081
MYD88	1.108	0.417	0.103	0.865~1.42
NMI	1.122	0.305	0.115	0.9~1.398
PLSCR1	1.058	0.617	0.056	0.848~1.32
PTX3	0.98	0.705	−0.02	0.883~1.088
PXDN	0.981	0.775	−0.02	0.858~1.121
PYGL	0.874	0.101	−0.134	0.745~1.026
RBBP8	0.912	0.468	−0.093	0.71~1.171
SERPINE1	0.958	0.506	−0.043	0.845~1.087
SOD2	0.836	0.042	−0.179	0.704~0.994
SRPX	0.946	0.218	−0.056	0.865~1.034
TENT5A	1.214	0.015	0.194	1.039~1.418
TGFBI	0.98	0.791	−0.02	0.847~1.135
TIMP1	1.079	0.454	0.076	0.884~1.318
VSIG4	1.004	0.977	0.004	0.77~1.309

All gene symbols were ordered alphabetically.

## Conclusions

The most comprehensive set of genome-scale mRNA expression data was constructed by combining GBM and normal control samples from 48 studies, resulting thousands of samples for generating robust genes signature. Based on large-scale gene expression data of GBMs, we have identified 147 robust differential expression genes, which showed the underlying gene expression level differences between NC and GBMs samples. Moreover, the most of identified robust DEGs (67%) were reported that closed to associated with GBM, which suggested high reproducibility with published papers. Furthermore, the GO term and KEGG pathway enrichment results based these robust DEGs may contribute to better understand the molecular mechanisms of GBM. More importantly, based on these robustness DEGs, three new hub genes including RFC4, ZWINT, and TYMS and three top transcriptional factors TATA, E2F4DP1 and HFH4, and two miRNA hsa-mir-519E and hsa-mir-527 were identified in the present study. Furthermore, survival analysis based on the TCGA GBM data revealed 38 genes expression significantly affect the prognosis of GBM in OS (p < 0.05). In sum, these hub genes, transcriptional factors and microRNAs may be potential molecular targets for therapies of GBMs.

## Electronic supplementary material

Supplementary Information
