# Supplementary material for: Genome-wide expression profiling of glioblastoma using a large combined cohort
Source: Sci Rep. 2018 Oct 10;8:15104. doi: 10.1038/s41598-018-33323-z (PMC6180049; doi:10.1038/s41598-018-33323-z)
Supplement: Supplementary file 1 — Supplementary Information [file 41598_2018_33323_MOESM1_ESM.pdf]

Supplementary Materials for:

Genome-wide expression profiling of glioblastoma using a large combined cohort

Jing Tang[1,2], Dian He[2,3]*, Pingrong Yang[2,3], Junquan He[2,3] and Yang Zhang[1,2]*

[1] Innovative Drug Research and Bioinformatics Group, School of Pharmaceutical Sciences and Innovative Drug Research Centre, Chongqing University, Chongqing 401331, China

[2] Materia Medica Development Group, Institute of Medicinal Chemistry, Lanzhou University School of Pharmacy, Lanzhou 730000, China

[3] Gansu Institute for Drug Control, Lanzhou 730070, China

* Corresponding author: Dian He (hed@lzu.edu.cn); Yang Zhang (20162901007@cqu.edu.cn)

Supplementary Table S1. The 100 robust differential expression genes (DEGs) between GBMs and normal samples are associated with the GBMs. All gene symbols were ordered alphabetically. NO indicated the gene was not reported to be differential expression in GBM samples; Yes indicated the gene was reported to be differential expression in GBM samples; Yes (UP) indicated the gene was reported to be differential expression and overexpression/upregulated in GBM samples; Yes (Down) indicated the gene was reported to be differential expression and downregulated in GBM samples.

Gene symbol	Descriptions of gene is associated with GBM	Differential expression	Ref
ABCA1	ABC transporter gene (ABCA1) is identified to be associated with drug resistance of glioblastoma tumors.	NO	[1]
ACKR3	ACKR3(CXCR7) mediates glioma cell migration and supports the development of therapeutic agents.	NO	[2]
ACTL6A	Actin like-6A promotes glioma progression through stabilization of transcriptional regulators YAP/TAZ.	Yes (UP)	[3]
AEBP1	AEBP1 is a transcription factor and identified that play role in survival of glioma cells.	Yes (UP)	[4]
ALOX5AP	ALOX5AP has reduced expression in glioma cell lines compared with normal brain.	Yes (Down)	[5]
ANXA1	ANXA1 may be involved in the progression of malignant gliomas.	Yes (UP)	[6]
BARD1	CLIC1 regarded as a modulator of cell cycle progression in human glioblastoma stem cells.	Yes (Down)	[7]
BCHE	Abnormal expressions of BCHE has also been observed in human glioma.	Yes	[8]
CD14	Lower expression CD14 in human glioblastoma is a potential implication for cerebral pathology.	Yes (Down)	[9]
CD163	CD163 genes was found to correlate with decreased survival for patients with primary GBM.	NO	[10]
CD44	Expression of CD44 correlated with hypoxia-induced gene signatures and poor survival.	Yes (Down)	[11]
CD58	CD58 has previously been shown to be highly efficacious in glioma abrogation.	NO	[12]
CD93	CD93 regarded as a key regulator of glioma angiogenesis and acting in cell adhesion.	NO	[13]
CDCA7L	It has been reported that CDCA7L is correlation to GBM patient survival time	NO	[14]
CENPK	CENPK is associated with tumor grades and TCGA subtypes as well as Ki-67 expression.	Yes	[15]
CHI3L2	CHI3L2(YKL-40) has increased expression in glioblastomas, and activates kinases ERK1/ERK2	Yes (UP)	[16]
CKS2	CKS2 gene expression levels were significantly higher in glioblastomas	Yes (UP)	[17]
CLIC1	CLIC1 was statistically significantly overexpressed in GBMs compared with normal brain tissues	Yes (UP)	[18]

CMTM3	CMTM3 promoted tumor cell invasion and identified be priority targets in glioblastomas	Yes (UP)	[19]
COL1A1	COL1A1 is useful in stratifying patients with GBM into subgroups for risk of recurrence at diagnosis.	Yes	[20]
COL1A2	COL1A2 is highly expressed genes in GBM spheroids as compared with normal brain	Yes (UP)	[21]
COL3A1	COL3A1 may be suitable biomarkers for diagnostic or therapeutic strategies for GBM	Yes (UP)	[22]
COL4A1	COL4A1 were found to be up-regulated and influential to development of the glioma	Yes (UP)	[23]
COL4A2	COL4A2 is novel candidate target gene that could potentially contribute to the pathogenesis of glioma	Yes (UP)	[24]
CRNDE	CRNDE expression was correlates with EGFR activation and modulates glioma cell growth	Yes (UP)	[25]
CTHRC1	CTHRC1 is overexpressed in glioblastoma tissues and cells	Yes (UP)	[26]
CXCR4	Glioma recurrence pattern is related to CXCR4 expression and a potential target for glioma therapy.	Yes	[27]
DSE	DSE promotes aggressive glioma cell phenotypes by enhancing HB-EGF/ErbB signaling.	Yes (UP)	[28]
DTX3L	DTX3L by siRNA transfection increased glioma cell apoptosis	Yes (UP)	[29]
EIF4EBP1	4E-BP1 regulates the sensitivity of glioma cells to chemotherapy through PI3K/Akt/mTOR pathway.	Yes (UP)	[30]
EMP1	EMP1 (novel MYC-interacting gene) were identified differentially expressed in gliomas.	Yes	[31]
EZH2	EZH2, is targeted by the MELK-FOXM1 complex. EZH2 can serve as a glioma therapeutic target.	NO	[32]
F2R	F2R encoding PAR1 was overexpressed, which, is causally associated with glioma progression.	Yes (UP)	[33]
FAM46A	FAM46A was up-regulated in GBM versus normal control and correlated with the OS of GBM.	Yes (UP)	[34]
FCER1G	FCER1G was upregulated in astrocytoma relative to normal cerebellum, and oligodendrogliomas.	Yes (UP)	[35]
FCGBP	Primary glioblastomas exhibited higher expression of extracellular response-associated gene FCGBP.	Yes (UP)	[36]
FNDC3B	microRNA-129-5p inhibit glioma cell proliferation, migration and invasiveness by targeting FNDC3B.	Yes (UP)	[37]
GABRA1	Upregulation of miR-155 in GBM could may downregulate GABRA1.	Yes (UP)	[38]
GBP1	Overexpression of GBP1 predicts poor prognosis and promotes tumor growth in human glioblastoma.	Yes (UP)	[39]
GBP2	GBP3 promotes glioma cell proliferation via SQSTM1/p62-ERK1/2 axis.	Yes (UP)	[40]
GBP3	GBP3 might represent as a new potential therapeutic target against glioma.	Yes (UP)	[40]
GJB6	GJB6 (Cx30) has the potential to influence growth,	NO	[41]

	proliferation and migration of GBM cells.		
GLIS3	Gliomas identified increased expression and gene amplification of GLIS3.	Yes (UP)	[42]
GPNMB	GPNMB was identified to promote the progression of brain glioblastoma via Na+/K+-ATPase.	Yes (UP)	[43]
GRM3	GRM3 promoter by transient transfection in both human neuroblastoma and astroglioma cell lines.	NO	[44]
HMOX1	HMOX1 expression in gliomas and its correlation with poor prognosis in patients with astrocytoma.	Yes (UP)	[45]
IFI44	IFI44 was demonstrated to be associated with CYR61 expression in glioma cells	Yes (UP)	[46]
IGFBP2	IGFBP2 is involved in immunosuppressive activities and is a potential immunotherapeutic target for GBM.	Yes (UP)	[47]
IGFBP3	MicroRNA-21 promotes glioma tumorigenesis by down-regulating IGFBP3.	Yes (UP)	[48]
KDELC2	The high expression of KDELC2 was negatively correlated with survival rates of glioma patients	Yes (UP)	[49]
KIAA0101	PCAF-mediated Akt1 acetylation enhances the proliferation of human glioblastoma cells.	NO	[50]
KRT222	KRT222 showed the strongest expression correlation with glioma tissues.	Yes	[49]
LY96	LY96 genetic regions related to innate immunity and risk of adult glioma.	NO	[51]
MCM2	MCM2 expression were of greater prognostic relevance than histological diagnosis.	Yes (UP)	[52]
MMP2	MMP2 was significantly increased by SDC1 overexpression and promoted glioma cell migration.	Yes (UP)	[53]
MS4A6A	MS4A6A was common events in glioma which could be used as novel biomarkers for prognosis of glioma.	NO	[54]
MTHFD2	MTHFD2 might play important roles and potentially be valuable in the prognosis and treatment of glioma	Yes (Down)	[55]
MYC	Myc have elevated expression in the glioma, and function in the proficient division of glioma cells.	Yes (UP)	[56]
MYD88	MyD88 deficiency or microglia depletion largely attenuated glioma expansion.	NO	[57]
NEFM	KLF6 inhibits the malignant phenotype of GBM in vitro and upregulates neuronal marker NEFM.	Yes (UP)	[58]
NEFH	miR-25 promotes GBMs cell proliferation and invasion by directly targeting NEFL.	NO	[59]
NID1	NID1 was identified to support proliferation of hESCs and diminish tumor load glioma.	NO	[60]
NMI	N-myc (and STAT) interactor (NMI) gene was reported in tumorigenesis, and in glioma progression.	NO	[61]
NNMT	NNMT is the most consistently overexpressed metabolism genes in glioma relative to normal brain.	Yes (UP)	[62]
NUSAP1	NUSAP1 is a potential prognosis marker for glioma patients and therapeutic strategies	Yes (UP)	[63]

PBK	PBK was candidate can be a promising molecular target for GBM treatment.	Yes (Down)	[64]
PHYHIP	PHYHIP is decreased expression level in glioma, compared to nor mal human brain.	Yes (Down)	[65]
PRRX1	Therapeutic targeting the PRRX1-DRD2-ERK/AKT axis is a promising strategy for treating GBMs.	Yes (UP)	[66]
PSMB8	PSMB8 can regulate glioma cell migration, proliferation, and apoptosis by PI3K/AKT pathways.	Yes (UP)	[67]
PTX3	Knockdown of PTX3 significantly decreases GBM8401 cell migration and invasion	Yes (Down)	[68]
PXDN	PXDN demonstrated varying degrees of higher expression in glioma specimens than normal tissues	Yes (UP)	[69]
PYGL	G6PC promotes glycogen accumulation by inhibiting PYGL and reduces cell migration and invasion.	NO	[70]
RBBP8	RBBP8 as a candidate oncogene involved in Rb-mediated cell cycle control.	NO	[71]
RBFOX1	Downregulated RBFOX1 is identified in GBMs compared with normal brain.	Yes (Down)	[72]
RGS1	Suppression of preinvasive RGS4 by mTOR inhibition optimizes glioma treatment.	NO	[73]
RNF135	RNF135 promotes the proliferation of glioblastoma cells in vivo and in vitro via the ERK pathway.	Yes (UP)	[74]
RRM2	BRCA1-mediated RRM2 expression protects GBM cells from endogenous replication stress	Yes (UP)	[75]
SERPINE1	SERPINE1, gene encode PAI-1 can promoted increased phosphorylation of STAT3 in glioma.	Yes (UP)	[76]
SLC12A5	SLC12A5 was identified to be downregulated expression in glioma compared with normal brain.	Yes (Down)	[77]
SLC2A10	Expression arrays reveal SLC2A10 gene play important role in the pathogenesis of glioblastoma.	Yes (UP)	[78]
SMC4	Overexpression of SMC4 activates TGFβ/Smad signaling, promotes aggressive phenotype in GBM.	Yes (UP)	[79]
SOD2	Specificity protein 1-modulated SOD2 can enhance temozolomide resistance in glioma.	Yes (UP)	[80]
SOX11	SOX11 expression was an indicator of favorable prognosis in glioma and expressed in gliomas.	Yes (UP)	[81]
SOX4	SOX4 inhibits glioma cell growth and induces G0/G1 cell cycle arrest through Akt-p53 axis.	Yes (UP)	[82]
SPRY1	IGFBP2 induces SPRY1 expression via NF-κB signaling pathway in glioblastoma.	Yes (UP)	[83]
SYNPR	SYNPR is downregulated differently expressed genes (DEGs) in GBM tissue samples.	Yes (Down)	[84]
TGFB1I1	TGFB1I1 is required for TGF-β stimulated EMT that contributes to progression of astrocytomas.	Yes (UP)	[85]
TGFBI	TGFBI is a potential prognostic molecule, which is correlates with glioma pathological grades.	Yes (UP)	[86]
TIMP1	The differential expression of individual TIMP1 may	Yes	[87]

	contribute to the pathophysiology of human GBM.		
TIMP4	The differential expression of individual TIMP4 may contribute to the pathophysiology of human GBM.	Yes	[87]
TMEM255A	TMEM255A is over-expressed in the differentiated GBM oncospheres.	Yes (UP)	[88]
TMEM45A	Suppressing of TMEM45A expression in glioma remarkably suppressed cell migration and invasion.	Yes (Down)	[89]
TNFSF13B	Target gene TNFSF13B could potentially contribute to the pathogenesis of glioma.	Yes (UP)	[24]
TOP2A	Over-expression of TOP2A as a prognostic biomarker in patients with GBM.	Yes (UP)	[90]
TPX2	TPX2 promotes glioma cell proliferation and invasion via activating the AKT signaling pathway.	Yes (UP)	[91]
UBE2C	Upregulated UBE2C gene was associated with the aggressive progression of GBM.	Yes (UP)	[31]
VCAM1	TNF may have significant effects on glioma biology by up-regulating VCAM-1.	Yes (UP)	[92]
VSIG4	Overexpressing VSIG4 can significantly promoted invasion and migration in glioblastoma.	Yes (UP)	[93]
WNT5A	WNT5A expression was in recurrent GBMs relative to matched intratumoral and primary GBMs.	Yes (UP)	[94]
ZNF217	ZNF217 suppresses cell growth, migration, and invasion of glioma cells in vitro.	NO	[95]

Supplementary Table S2. The top 10 significantly enriched transcript factors based on 1% differentially-expressed genes between glioblastoma and normal control samples. The significance level was set at false discovery rate (FDR) ≤ 0.05. UN suggested the transcript factor has not been reported in current GBM-associated studies.

**Transcript factor**	**GBMs-associated descriptions**	**FDR**	**Reference**
ICSBP_Q6	ICSBP, is also known as interferon regulatory factor-8 (IRF-8), which possibly involved in hypoxia-modulated CCL4 expression of macrophages and promoted glioblastoma invasion.	6.77E-09	[96]
STTTCRNTTT_IRF_Q6	Down regulation of IRF1 by shRNA blocked autophagy and increased AIF-dependent apoptosis in bevacizumab-treated glioma cells.	1.02E-06	[96,97]
TATAAA_TATA_01	UN	1.26E-06	
CTTTGT_LEF1_Q2	LEF1 regulates glioblastoma cell proliferation, migration, invasion, and cancer stem-like cell self-renewal.	4.95E-04	[98]
E2F_Q3_01	E2F transcription factors associated with up-regulated genes in glioblastoma	4.96E-04	[99]
E2F4DP1_01	UN	4.96E-04	
E2F_Q6_01	E2F transcription factors associated with up-regulated genes in glioblastoma	4.96E-04	[99]
DBP_Q6	DBP associated with an increased risk of high-grade glioma	6.96E-04	[100]
NRSF_01	Inhibition of REST /NRSF suppresses proliferation and migration of glioblastoma cells	2.30E-07	[101]
PR_Q2	UN	9.34E-04	
YTATTTTNR_MEF2_02	Hypermethylation at C14 locus and downregulation of MEF2 are identified in GBM samples,	1.41E-03	[102]
TTGTTT_ FOXO4_01	Akt activation is significantly correlated with FOXO4, which resulted in deregulated phosphatidylinositol 3'-kinase (PI3K) pathway in glioblastoma patients	2.12E-03	[103]
CAGGTG_E12_Q6	UN	6.02E-03	
FOXD3_01	Down-regulation of long non-coding RNA FOXD3 antisense RNA 1 (FOXD3-AS1) inhibits cell proliferation, migration, and invasion in malignant glioma cells.	6.02E-03	[104]
HFH4_01	UN	6.02E-03	

Supplementary Table S3. The significantly enriched target miRNAs based on 1% differentially-expressed genes between glioblastoma and normal control samples. The significance level was set at false discovery rate (FDR) ⩽ 0.05. There are not significantly enrichment target miRNAs in down-regulated DEGs at a false discovery rate of 0.05. UN suggested the miRNA has not been reported in current GBM-associated studies.

Target miRNAs	GBMs-associated descriptions	FDR	Reference
ACTACCT_MIR196A_MIR196B	miR-196b is upregulated in glioma as compared with normal brain tissue and associated with regulation of cell proliferation	1.01E-03	[105]
ACCAAAG_MIR9	miR9 is a therapeutic target, which governs metastasis and thus determines prognosis in GBM through MAPKAP signaling	1.90E-03	[106]
GTGCAAT_MIR25_MIR32_MIR92_MIR363_MIR367	Upregulated miR-363 increases glioblastoma cell survival and proliferation.	2.82E-03	[107,108]
CTACCTC_LET7A_LET7B_LET7C_LET7D_LET7E_LET7F_MIR98_LET7G_LET7I	miR-98 is frequently downregulated in glioma tissues and cell lines	8.79E-03	[109]
CTTTGTA_MIR524	miR-524-5p behaves as a tumor suppressor by negatively targeting Jagged-1 and inhibits this oncogene in gliomas.	1.19E-02	[110]
CTTGTAT_MIR381	miR-381 can increase proliferation of glioma cells through the ERK and AKT signaling	1.19E-02	[111]
GCACTTT_MIR175P_MIR20A_MIR106A_MIR106B_MIR20B_MIR519D	Down-regulation of miR-106b suppresses the growth of human glioma cells<br>miR-106a enhances the invasiveness of human glioma stem cells by directly targeting TIMP-2	1.19E-02	[112,113]
GGCACTT_MIR519E	UN	1.37E-02	
CTTTGCA_MIR527	UN	1.53E-02	
AAGCACT_MIR520F	miR-520f targeting PDGFRB is downregulated in glioblastoma microvascular proliferation	1.53E-02	[114]
TGGTGCT_MIR29A_MIR29B_MIR29C	Overexpression of miR-29a reduces the oncogenic properties of glioblastoma stem cells	1.62E-02	[115]
TTGCACT_MIR130A_MIR301_MIR130B	miR-130a can predict response to temozolomide in patients	1.62E-02	[116]

	with glioblastoma multiforme		
TGCACTT_MIR519C_MIR519B_MIR519A	miR-519a functions as a tumor suppressor in glioma by targeting the oncogenic STAT3 pathway	2.08E-02	[117]
CAATGCA_MIR33	miR-33a promotes Glioma-initiating cell self-renewal via PKA and NOTCH pathways	3.30E-02	[118]
TTTGTAG_MIR520D	UN	3.71E-02	[119]

Supplementary Figure S1. The PPI network was constructed based on 1% differentially-expressed genes between glioblastoma and normal control samples by STRING tool, where nodes are proteins; edges represent protein-protein associations including known interactions (curated databases and experimentally determined) and predicted interactions (gene neighborhood and gene co-occurrence). The edge may be drawn with up to five differently colored lines, which indicate the existence of the five types of evidence used in protein-protein associations. A red line, gene fusions evidence; a green line, gene neighborhood evidence; a blue line, gene co-occurrence evidence; a light blue line, curated database evidence; a purple line, experimental evidence

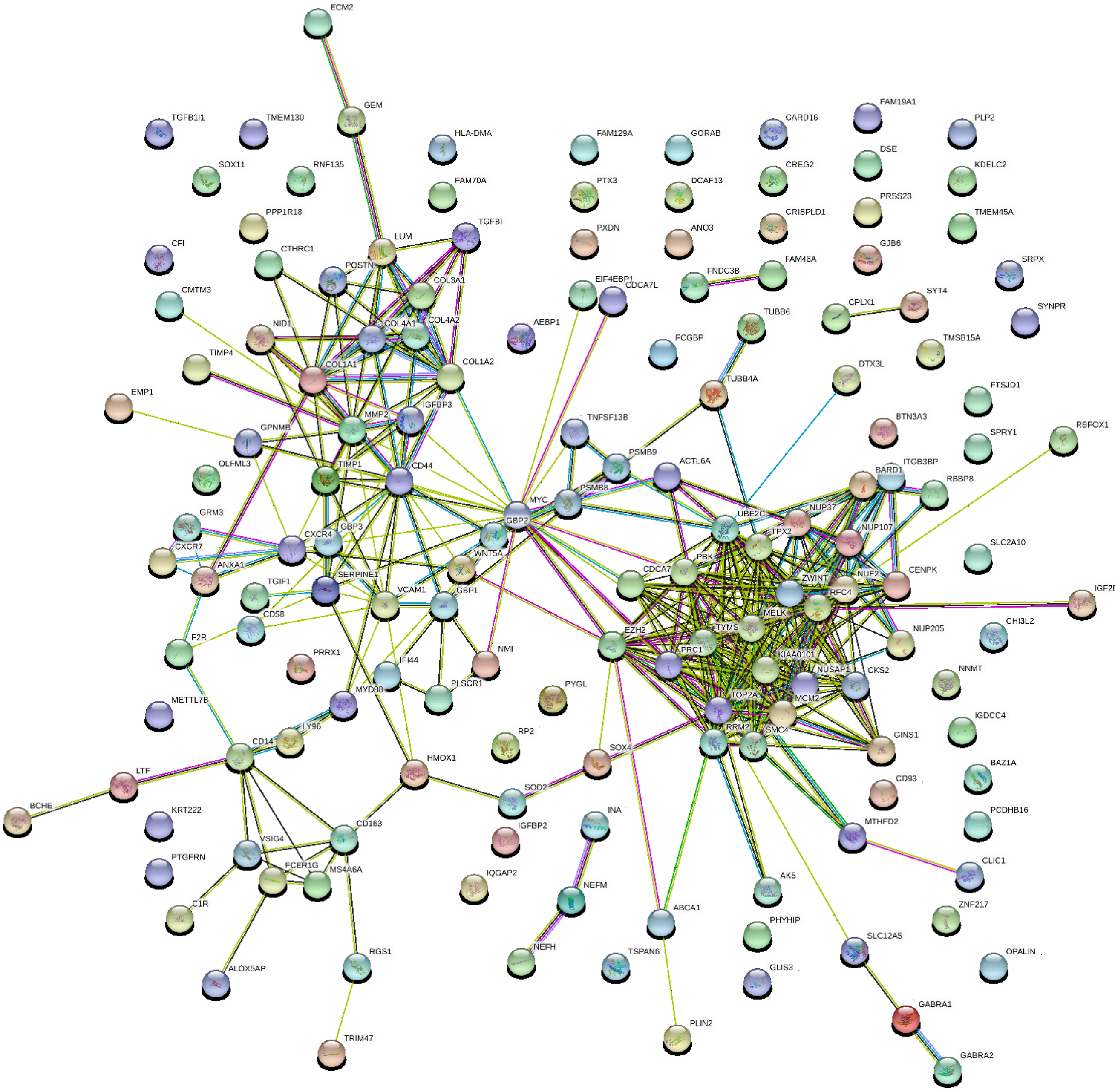

Supplementary Figure S2. Univariate survival analysis in GBM stratified by robust differential expression gene expression based on the TCGA data as determined by Kaplan-Meier estimates. 521 GBM cases with full data of both clinical and gene expression were collected from the TCGA database. Kaplan-Meier estimates (log-rank test) were made and found 38 genes expression were significantly affect the prognosis of GBM in OS ($p<0.05$)

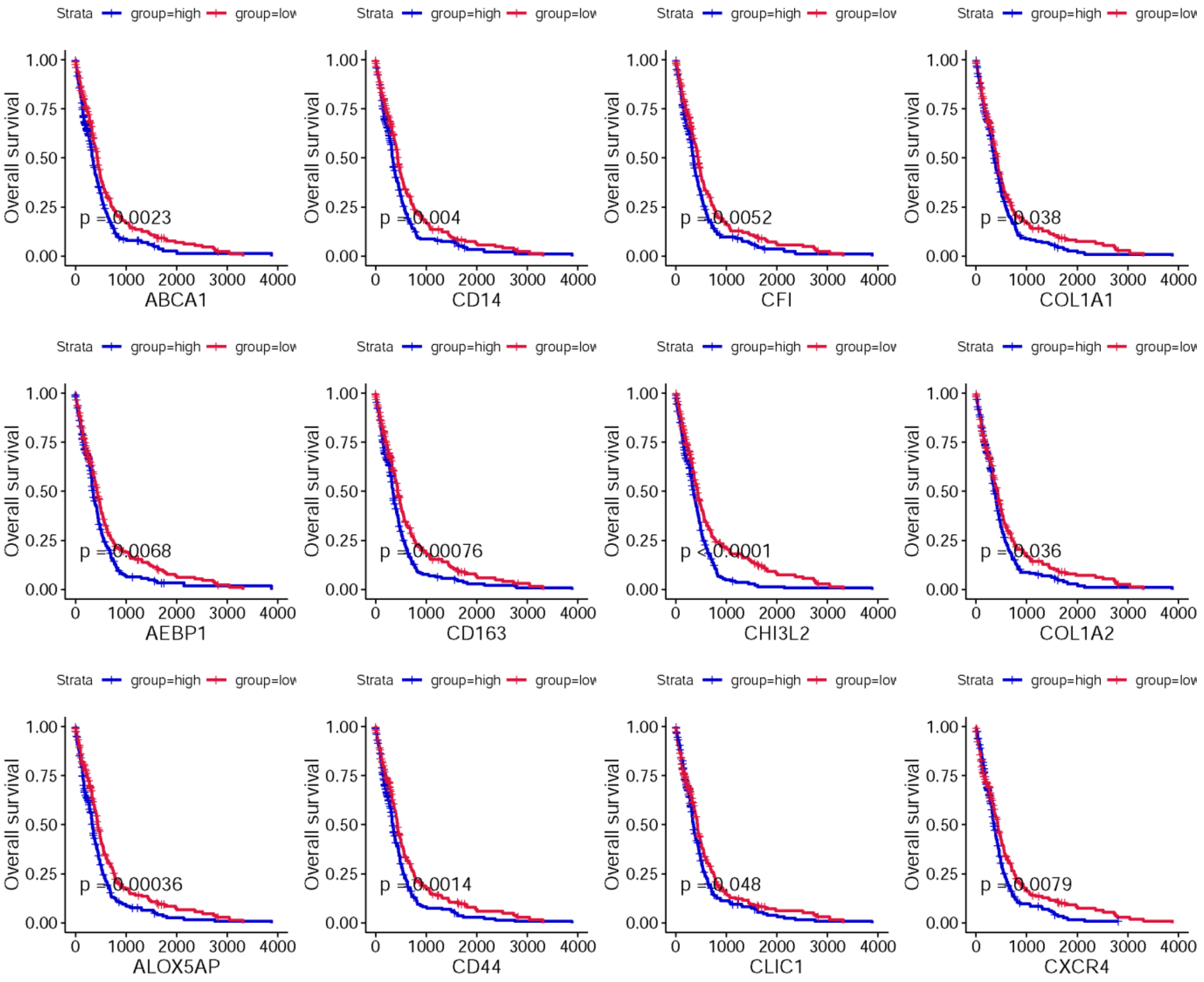

Supplementary Figure S2 (continued). Univariate survival analysis in GBM stratified by robust differential expression gene expression based on the TCGA data as determined by Kaplan-Meier estimates. 521 GBM cases with full data of both clinical and gene expression were collected from the TCGA database. Kaplan-Meier estimates (log-rank test) were made and found 38 genes expression were significantly affect the prognosis of GBM in OS (p<0.05)

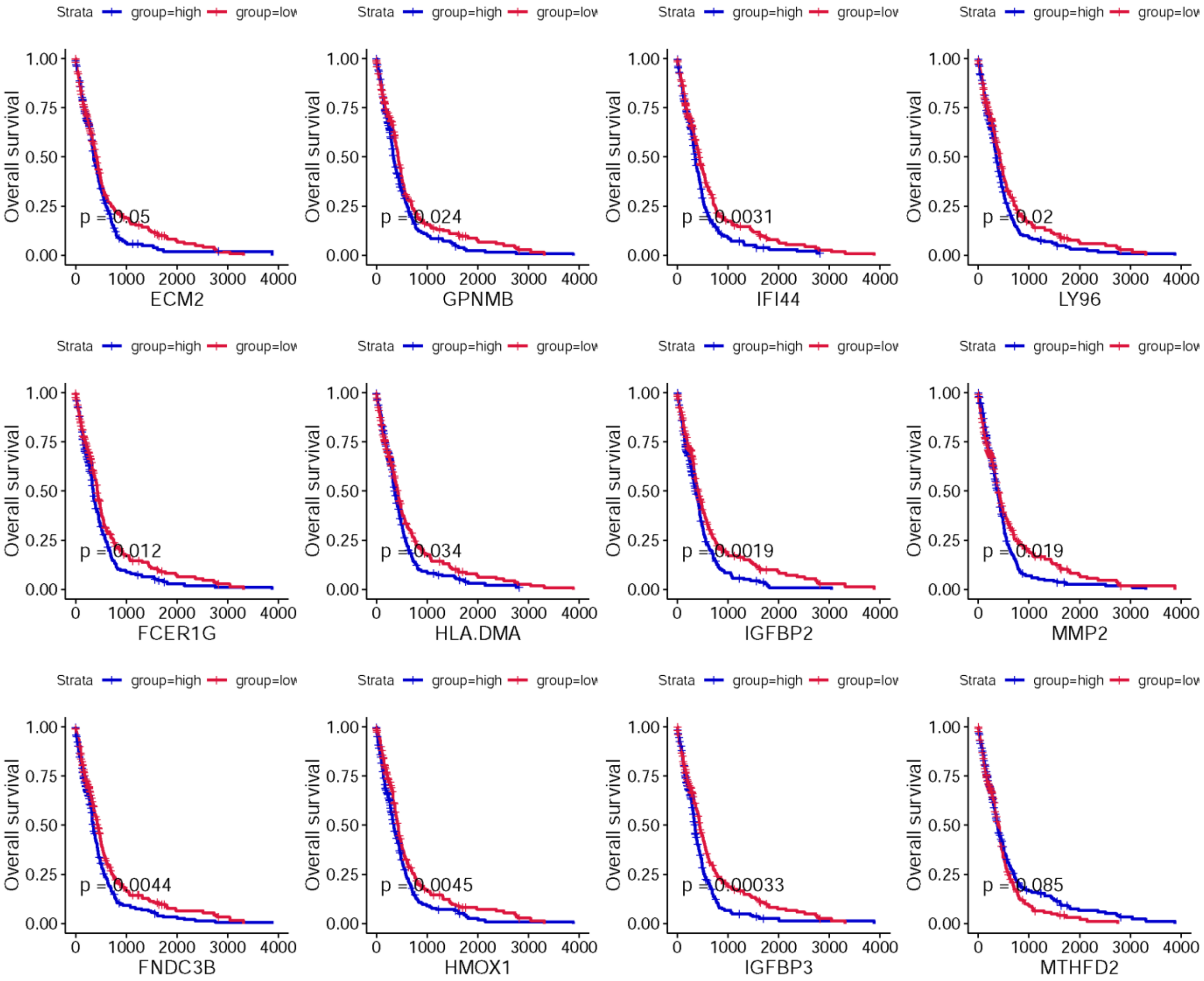

Supplementary Figure S2 (continued). Univariate survival analysis in GBM stratified by robust differential expression gene expression based on the TCGA data as determined by Kaplan-Meier estimates. 521 GBM cases with full data of both clinical and gene expression were collected from the TCGA database. Kaplan-Meier estimates (log-rank test) were made and found 38 genes expression were significantly affect the prognosis of GBM in OS (p<0.05)

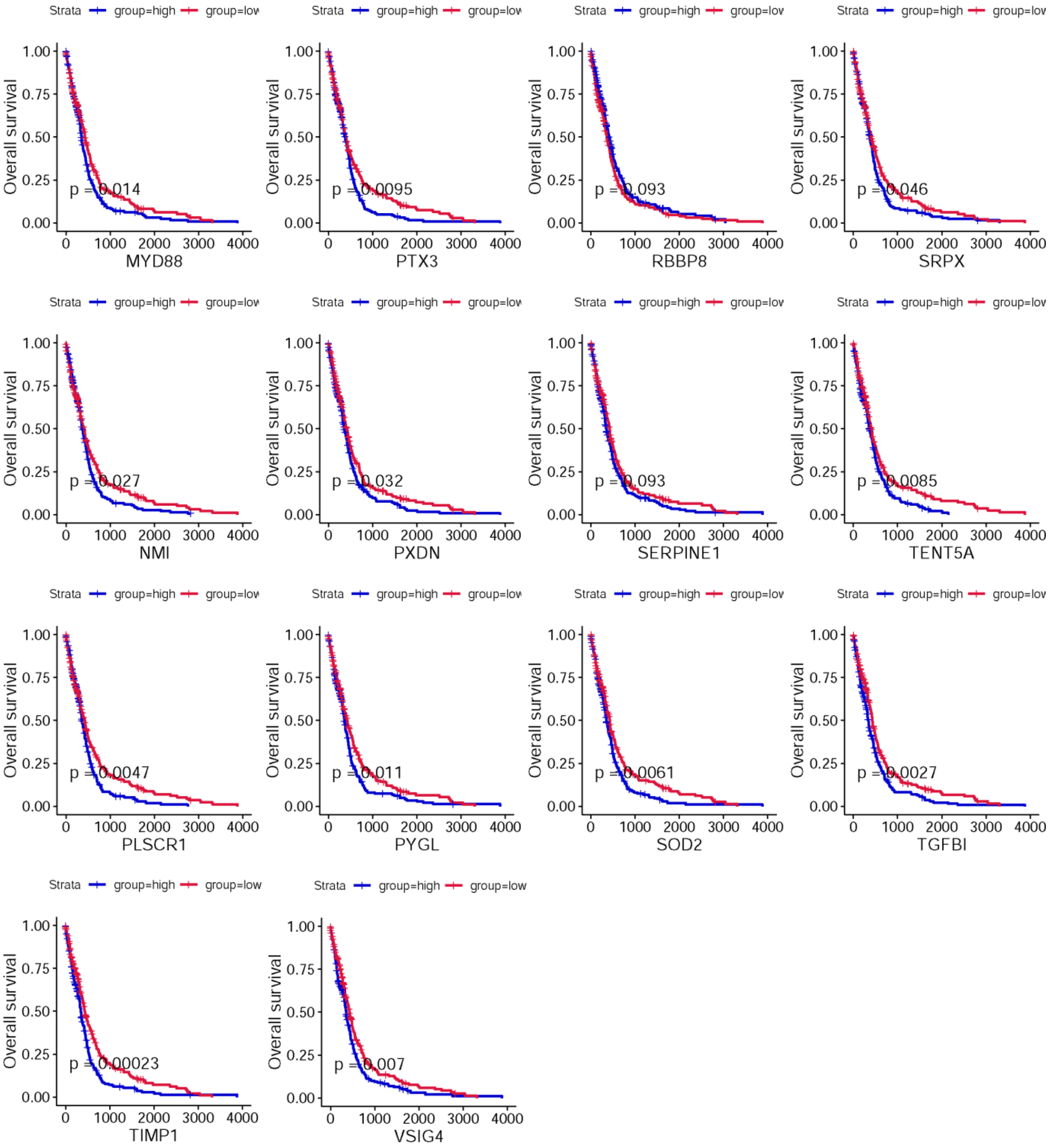

Supplementary Figure S3. Multivariate survival analysis using Cox's regression mode in GBM stratified by risk scores.

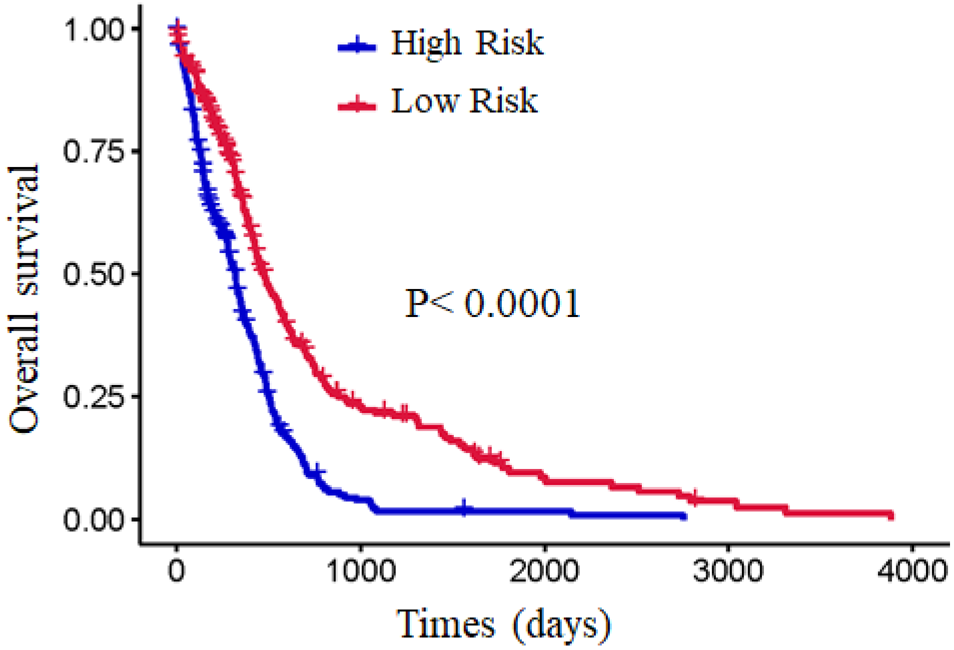

References

1. Gadaleta F, Bessonov K, Van Steen K (2017) Integration of gene expression and methylation to unravel biological networks in glioblastoma patients. Genetic epidemiology 41 (2):136-144. doi:10.1002/gepi.22028
2. Esencay M, Sarfraz Y, Zagzag D (2013) CXCR7 is induced by hypoxia and mediates glioma cell migration towards SDF-1alpha. BMC cancer 13:347. doi:10.1186/1471-2407-13-347
3. Ji J, Xu R, Zhang X, Han M, Xu Y, Wei Y, Ding K, Wang S, Bin H, Chen A, Di Z, Jiang Z, Xu S, Zhang Q, Li W, Ni S, Wang J, Li X (2018) Actin like-6A promotes glioma progression through stabilization of transcriptional regulators YAP/TAZ. Cell death & disease 9 (5):517. doi:10.1038/s41419-018-0548-3
4. Ladha J, Sinha S, Bhat V, Donakonda S, Rao SM (2012) Identification of genomic targets of transcription factor AEBP1 and its role in survival of glioma cells. Molecular cancer research : MCR 10 (8):1039-1051. doi:10.1158/1541-7786.MCR-11-0488
5. Saigusa K, Imoto I, Tanikawa C, Aoyagi M, Ohno K, Nakamura Y, Inazawa J (2007) RGC32, a novel p53-inducible gene, is located on centrosomes during mitosis and results in G2/M arrest. Oncogene 26 (8):1110-1121. doi:10.1038/sj.onc.1210148
6. Xu Y, Wang J, Xu Y, Xiao H, Li J, Wang Z (2017) Screening critical genes associated with malignant glioma using bioinformatics analysis. Molecular medicine reports 16 (5):6580-6589. doi:10.3892/mmr.2017.7471
7. Gritti M, Wurth R, Angelini M, Barbieri F, Peretti M, Pizzi E, Pattarozzi A, Carra E, Sirito R, Daga A, Curmi PM, Mazzanti M, Florio T (2014) Metformin repositioning as antitumoral agent: selective antiproliferative effects in human glioblastoma stem cells, via inhibition of CLIC1-mediated ion current. Oncotarget 5 (22):11252-11268. doi:10.18632/oncotarget.2617
8. Shan L (2004) Near-infrared dye IRDye 800CW-labeled butyrylcholinesterase. In: Molecular Imaging and Contrast Agent Database (MICAD). Bethesda (MD),
9. Haghparast A, Heidari Kharaji M, Malvandi AM (2011) Down-regulation of CD14 transcripts in human glioblastoma cell line U87 MG. Iranian journal of immunology : IJI 8 (2):111-119. doi:IJIv8i2A7
10. Ge H, Mu L, Jin L, Yang C, Chang YE, Long Y, DeLeon G, Deleyrolle L, Mitchell DA, Kubilis PS, Lu D, Qi J, Gu Y, Lin Z, Huang J (2017) Tumor associated CD70 expression is involved in promoting tumor migration and macrophage infiltration in GBM. International journal of cancer 141 (7):1434-1444. doi:10.1002/ijc.30830
11. Pietras A, Katz AM, Ekstrom EJ, Wee B, Halliday JJ, Pitter KL, Werbeck JL, Amankulor NM, Huse JT, Holland EC (2014) Osteopontin-CD44 signaling in the glioma perivascular niche enhances cancer stem cell phenotypes and promotes aggressive tumor growth. Cell stem cell 14 (3):357-369. doi:10.1016/j.stem.2014.01.005
12. Chaudhuri S, Singh MK, Bhattacharya D, Acharya S, Chatterjee S, Kumar P, Bhattacharjee P, Basu AK, Sa G, Das T, Ghosh TK, Chaudhuri S (2014) The novel immunotherapeutic molecule T11TS modulates glioma-induced changes of key components of the immunological synapse in favor of T cell activation and glioma abrogation. Journal of neuro-oncology 120 (1):19-31. doi:10.1007/s11060-014-1528-9
13. Langenkamp E, Zhang L, Lugano R, Huang H, Elhassan TE, Georganaki M, Bazzar W, Loof J, Trendelenburg G, Essand M, Ponten F, Smits A, Dimberg A (2015) Elevated expression of the C-type lectin CD93 in the glioblastoma vasculature regulates cytoskeletal rearrangements that enhance vessel function and reduce host survival. Cancer research 75 (21):4504-4516. doi:10.1158/0008-5472.CAN-14-3636
14. Amlin-Van Schaick JC, Kim S, DiFabio C, Lee MH, Broman KW, Reilly KM (2012) Arlm1 is a male-specific modifier of astrocytoma resistance on mouse Chr 12. Neuro-oncology 14 (2):160-174. doi:10.1093/neuonc/nor206
15. Liu Y, Hu H, Zhang C, Wang H, Zhang W, Wang Z, Li M, Zhang W, Zhou D, Jiang T (2015) Co-expression of mitosis-regulating genes contributes to malignant progression and prognosis in oligodendrogliomas. Oncotarget 6 (35):38257-38269. doi:10.18632/oncotarget.5499
16. Areshkov PA, Kavsan VM (2010) Chitinase 3-like protein 2 (CHI3L2, YKL-39) activates phosphorylation

of extracellular signal-regulated kinases ERK1/ERK2 in human embryonic kidney (HEK293) and human glioblastoma (U87 MG) cells. TSitologiia i genetika 44 (1):3-9

17. Scrideli CA, Carlotti CG, Jr., Okamoto OK, Andrade VS, Cortez MA, Motta FJ, Lucio-Eterovic AK, Neder L, Rosemberg S, Oba-Shinjo SM, Marie SK, Tone LG (2008) Gene expression profile analysis of primary glioblastomas and non-neoplastic brain tissue: identification of potential target genes by oligonucleotide microarray and real-time quantitative PCR. Journal of neuro-oncology 88 (3):281-291. doi:10.1007/s11060-008-9579-4
18. Setti M, Savalli N, Osti D, Richichi C, Angelini M, Brescia P, Fornasari L, Carro MS, Mazzanti M, Pelicci G (2013) Functional role of CLIC1 ion channel in glioblastoma-derived stem/progenitor cells. Journal of the National Cancer Institute 105 (21):1644-1655. doi:10.1093/jnci/djt278
19. Delic S, Thuy A, Schulze M, Proescholdt MA, Dietrich P, Bosserhoff AK, Riemenschneider MJ (2015) Systematic investigation of CMTM family genes suggests relevance to glioblastoma pathogenesis and CMTM1 and CMTM3 as priority targets. Genes, chromosomes & cancer 54 (7):433-443. doi:10.1002/gcc.22255
20. Balbous A, Cortes U, Guilloteau K, Villalva C, Flamant S, Gaillard A, Milin S, Wager M, Sorel N, Guilhot J, Bennaceur-Griscelli A, Turhan A, Chomel JC, Karayan-Tapon L (2014) A mesenchymal glioma stem cell profile is related to clinical outcome. Oncogenesis 3:e91. doi:10.1038/oncsis.2014.5
21. Ernst A, Hofmann S, Ahmadi R, Becker N, Korshunov A, Engel F, Hartmann C, Felsberg J, Sabel M, Peterziel H, Durchdewald M, Hess J, Barbus S, Campos B, Starzinski-Powitz A, Unterberg A, Reifenberger G, Lichter P, Herold-Mende C, Radlwimmer B (2009) Genomic and expression profiling of glioblastoma stem cell-like spheroid cultures identifies novel tumor-relevant genes associated with survival. Clinical cancer research : an official journal of the American Association for Cancer Research 15 (21):6541-6550. doi:10.1158/1078-0432.CCR-09-0695
22. Gao YF, Mao XY, Zhu T, Mao CX, Liu ZX, Wang ZB, Li L, Li X, Yin JY, Zhang W, Zhou HH, Liu ZQ (2016) COL3A1 and SNAP91: novel glioblastoma markers with diagnostic and prognostic value. Oncotarget 7 (43):70494-70503. doi:10.18632/oncotarget.12038
23. Kunkle BW, Yoo C, Roy D (2013) Reverse engineering of modified genes by Bayesian network analysis defines molecular determinants critical to the development of glioblastoma. PloS one 8 (5):e64140. doi:10.1371/journal.pone.0064140
24. Ruano Y, Mollejo M, Ribalta T, Fiano C, Camacho FI, Gomez E, de Lope AR, Hernandez-Moneo JL, Martinez P, Melendez B (2006) Identification of novel candidate target genes in amplicons of Glioblastoma multiforme tumors detected by expression and CGH microarray profiling. Molecular cancer 5:39. doi:10.1186/1476-4598-5-39
25. Kiang KM, Zhang XQ, Zhang GP, Li N, Cheng SY, Poon MW, Pu JK, Lui WM, Leung GK (2017) CRNDE Expression Positively Correlates with EGFR Activation and Modulates Glioma Cell Growth. Targeted oncology 12 (3):353-363. doi:10.1007/s11523-017-0488-3
26. Liu J, Li W, Liu S, Zheng X, Shi L, Zhang W, Yang H (2017) Knockdown of Collagen Triple Helix Repeat Containing 1 (CTHRC1) Inhibits Epithelial-Mesenchymal Transition and Cellular Migration in Glioblastoma Cells. Oncology research 25 (2):225-232. doi:10.3727/096504016X14732772150587
27. Tang W, Wang X, Chen Y, Zhang J, Chen Y, Lin Z (2015) CXCL12 and CXCR4 as predictive biomarkers of glioma recurrence pattern after total resection. Pathologie-biologie 63 (4-5):190-198. doi:10.1016/j.patbio.2015.07.002
28. Liao WC, Liao CK, Tsai YH, Tseng TJ, Chuang LC, Lan CT, Chang HM, Liu CH (2018) DSE promotes aggressive glioma cell phenotypes by enhancing HB-EGF/ErbB signaling. PloS one 13 (6):e0198364. doi:10.1371/journal.pone.0198364
29. Xu P, Tao X, Zhao C, Huang Q, Chang H, Ban N, Bei Y, Xia X, Shen C, Wang K, Xu L, Wu P, Ren J, Wang D (2017) DTX3L is upregulated in glioma and is associated with glioma progression. International journal of

molecular medicine 40 (2):491-498. doi:10.3892/ijmm.2017.3023
30. Zhu HL, Xie SM, Fang M, Zhang JJ, Weng ZP, Zhong XY (2014) 4E-BP1 regulates the sensitivity of human glioma cells to chemotherapy through PI3K/Akt/mTOR-independent pathway. Neuropathology : official journal of the Japanese Society of Neuropathology 34 (3):227-235. doi:10.1111/neup.12085
31. Bredel M, Bredel C, Juric D, Harsh GR, Vogel H, Recht LD, Sikic BI (2005) Functional network analysis reveals extended gliomagenesis pathway maps and three novel MYC-interacting genes in human gliomas. Cancer research 65 (19):8679-8689. doi:10.1158/0008-5472.CAN-05-1204
32. Kim SH, Joshi K, Ezhilarasan R, Myers TR, Siu J, Gu C, Nakano-Okuno M, Taylor D, Minata M, Sulman EP, Lee J, Bhat KP, Salcini AE, Nakano I (2015) EZH2 protects glioma stem cells from radiation-induced cell death in a MELK/FOXM1-dependent manner. Stem cell reports 4 (2):226-238. doi:10.1016/j.stemcr.2014.12.006
33. Auvergne R, Wu C, Connell A, Au S, Cornwell A, Osipovitch M, Benraiss A, Dangelmajer S, Guerrero-Cazares H, Quinones-Hinojosa A, Goldman SA (2016) PAR1 inhibition suppresses the self-renewal and growth of A2B5-defined glioma progenitor cells and their derived gliomas in vivo. Oncogene 35 (29):3817-3828. doi:10.1038/onc.2015.452
34. Xiong J, Bing Z, Su Y, Deng D, Peng X (2014) An integrated mRNA and microRNA expression signature for glioblastoma multiforme prognosis. PloS one 9 (5):e98419. doi:10.1371/journal.pone.0098419
35. Huang H, Hara A, Homma T, Yonekawa Y, Ohgaki H (2005) Altered expression of immune defense genes in pilocytic astrocytomas. Journal of neuropathology and experimental neurology 64 (10):891-901
36. Rad A, Sorbye SW, Dreyer G, Hovland S, Falang BM, Louw M, Skjeldestad FE (2017) HPV types in cervical cancer tissue in South Africa: A head-to-head comparison by mRNA and DNA tests. Medicine 96 (47):e8752. doi:10.1097/MD.0000000000008752
37. Xu H, Hu Y, Qiu W (2017) Potential mechanisms of microRNA-129-5p in inhibiting cell processes including viability, proliferation, migration and invasiveness of glioblastoma cells U87 through targeting FNDC3B. Biomedicine & pharmacotherapy = Biomedecine & pharmacotherapie 87:405-411. doi:10.1016/j.biopha.2016.12.100
38. D'Urso PI, D'Urso OF, Storelli C, Mallardo M, Gianfreda CD, Montinaro A, Cimmino A, Pietro C, Marsigliante S (2012) miR-155 is up-regulated in primary and secondary glioblastoma and promotes tumour growth by inhibiting GABA receptors. International journal of oncology 41 (1):228-234. doi:10.3892/ijo.2012.1420
39. Ji X, Zhu H, Dai X, Xi Y, Sheng Y, Gao C, Liu H, Xue Y, Liu J, Shi J, Zhang Y, Chen Y, Dai X, Li M, Wang A, Dong J (2018) Overexpression of GBP1 predicts poor prognosis and promotes tumor growth in human glioblastoma multiforme. Cancer biomarkers : section A of Disease markers. doi:10.3233/CBM-171177
40. Xu H, Sun L, Zheng Y, Yu S, Ou-Yang J, Han H, Dai X, Yu X, Li M, Lan Q (2018) GBP3 promotes glioma cell proliferation via SQSTM1/p62-ERK1/2 axis. Biochemical and biophysical research communications 495 (1):446-453. doi:10.1016/j.bbrc.2017.11.050
41. Arun S, Vanisree AJ, Ravisankar S (2016) Connexin 30 downregulates Insulin-like growth factor receptor-1, abolishes Erk and potentiates effects of an IGF-R inhibitor in a glioma cell line. Brain research 1643:80-90. doi:10.1016/j.brainres.2016.04.061
42. Cooper LA, Gutman DA, Long Q, Johnson BA, Cholleti SR, Kurc T, Saltz JH, Brat DJ, Moreno CS (2010) The proneural molecular signature is enriched in oligodendrogliomas and predicts improved survival among diffuse gliomas. PloS one 5 (9):e12548. doi:10.1371/journal.pone.0012548
43. Ono Y, Chiba S, Yano H, Nakayama N, Saio M, Tsuruma K, Shimazawa M, Iwama T, Hara H (2016) Glycoprotein nonmetastatic melanoma protein B (GPNMB) promotes the progression of brain glioblastoma via Na(+)/K(+)-ATPase. Biochemical and biophysical research communications 481 (1-2):7-12. doi:10.1016/j.bbrc.2016.11.034
44. Corti C, Xuereb JH, Corsi M, Ferraguti F (2001) Identification and characterization of the promoter region

of the GRM3 gene. Biochemical and biophysical research communications 286 (2):381-387. doi:10.1006/bbrc.2001.5391
45. Gandini NA, Fermento ME, Salomon DG, Obiol DJ, Andres NC, Zenklusen JC, Arevalo J, Blasco J, Lopez Romero A, Facchinetti MM, Curino AC (2014) Heme oxygenase-1 expression in human gliomas and its correlation with poor prognosis in patients with astrocytoma. Tumour biology : the journal of the International Society for Oncodevelopmental Biology and Medicine 35 (3):2803-2815. doi:10.1007/s13277-013-1373-z
46. Wang R, Wei B, Wei J, Tian Y, Du C (2017) Cysteine-rich 61-associated gene expression profile alterations in human glioma cells. Molecular medicine reports 16 (4):5561-5567. doi:10.3892/mmr.2017.7216
47. Cai J, Chen Q, Cui Y, Dong J, Chen M, Wu P, Jiang C (2018) Immune heterogeneity and clinicopathologic characterization of IGFBP2 in 2447 glioma samples. Oncoimmunology 7 (5):e1426516. doi:10.1080/2162402X.2018.1426516
48. Yang CH, Yue J, Pfeffer SR, Fan M, Paulus E, Hosni-Ahmed A, Sims M, Qayyum S, Davidoff AM, Handorf CR, Pfeffer LM (2014) MicroRNA-21 promotes glioblastoma tumorigenesis by down-regulating insulin-like growth factor-binding protein-3 (IGFBP3). The Journal of biological chemistry 289 (36):25079-25087. doi:10.1074/jbc.M114.593863
49. Liang A, Zhou B, Sun W (2017) Integrated genomic characterization of cancer genes in glioma. Cancer cell international 17:90. doi:10.1186/s12935-017-0458-y
50. Zhang S, Sun G, Wang Z, Wan Y, Guo J, Shi L (2015) PCAF-mediated Akt1 acetylation enhances the proliferation of human glioblastoma cells. Tumour biology : the journal of the International Society for Oncodevelopmental Biology and Medicine 36 (3):1455-1462. doi:10.1007/s13277-014-2522-8
51. Rajaraman P, Brenner AV, Butler MA, Wang SS, Pfeiffer RM, Ruder AM, Linet MS, Yeager M, Wang Z, Orr N, Fine HA, Kwon D, Thomas G, Rothman N, Inskip PD, Chanock SJ (2009) Common variation in genes related to innate immunity and risk of adult glioma. Cancer epidemiology, biomarkers & prevention : a publication of the American Association for Cancer Research, cosponsored by the American Society of Preventive Oncology 18 (5):1651-1658. doi:10.1158/1055-9965.EPI-08-1041
52. Hua C, Zhao G, Li Y, Bie L (2014) Minichromosome Maintenance (MCM) Family as potential diagnostic and prognostic tumor markers for human gliomas. BMC cancer 14:526. doi:10.1186/1471-2407-14-526
53. Chen J, Tang J, Chen W, Gao Y, He Y, Zhang Q, Ran Q, Cao F, Yao S (2017) Effects of syndecan-1 on the expression of syntenin and the migration of U251 glioma cells. Oncology letters 14 (6):7217-7224. doi:10.3892/ol.2017.7170
54. Ma J, Hou X, Li M, Ren H, Fang S, Wang X, He C (2015) Genome-wide methylation profiling reveals new biomarkers for prognosis prediction of glioblastoma. Journal of cancer research and therapeutics 11 Suppl 2:C212-215. doi:10.4103/0973-1482.168188
55. Liu M, Xu Z, Du Z, Wu B, Jin T, Xu K, Xu L, Li E, Xu H (2017) The Identification of Key Genes and Pathways in Glioma by Bioinformatics Analysis. Journal of immunology research 2017:1278081. doi:10.1155/2017/1278081
56. Annibali D, Whitfield JR, Favuzzi E, Jauset T, Serrano E, Cuartas I, Redondo-Campos S, Folch G, Gonzalez-Junca A, Sodir NM, Masso-Valles D, Beaulieu ME, Swigart LB, Mc Gee MM, Somma MP, Nasi S, Seoane J, Evan GI, Soucek L (2014) Myc inhibition is effective against glioma and reveals a role for Myc in proficient mitosis. Nature communications 5:4632. doi:10.1038/ncomms5632
57. Markovic DS, Vinnakota K, Chirasani S, Synowitz M, Raguet H, Stock K, Sliwa M, Lehmann S, Kalin R, van Rooijen N, Holmbeck K, Heppner FL, Kiwit J, Matyash V, Lehnardt S, Kaminska B, Glass R, Kettenmann H (2009) Gliomas induce and exploit microglial MT1-MMP expression for tumor expansion. Proceedings of the National Academy of Sciences of the United States of America 106 (30):12530-12535. doi:10.1073/pnas.0804273106
58. Masilamani AP, Ferrarese R, Kling E, Thudi NK, Kim H, Scholtens DM, Dai F, Hadler M, Unterkircher T,

Platania L, Weyerbrock A, Prinz M, Gillespie GY, Harsh GI, Bredel M, Carro MS (2017) KLF6 depletion promotes NF-kappaB signaling in glioblastoma. Oncogene 36 (25):3562-3575. doi:10.1038/onc.2016.507
59. Peng G, Yuan X, Yuan J, Liu Q, Dai M, Shen C, Ma J, Liao Y, Jiang W (2015) miR-25 promotes glioblastoma cell proliferation and invasion by directly targeting NEFL. Molecular and cellular biochemistry 409 (1-2):103-111. doi:10.1007/s11010-015-2516-x
60. Pinney E, Zimber M, Schenone A, Montes-Camacho M, Ziegler F, Naughton GK (2011) Human Embryonic-like ECM (hECM) Stimulates Proliferation and Differentiation in Stem Cells While Killing Cancer Cells. International journal of stem cells 4 (1):70-75
61. Meng D, Li X, Zhang S, Zhao Y, Song X, Chen Y, Wang S, Mao Y, Chen H, Lu D (2015) Genetic variants in N-myc (and STAT) interactor and susceptibility to glioma in a Chinese Han population. Tumour biology : the journal of the International Society for Oncodevelopmental Biology and Medicine 36 (3):1579-1588. doi:10.1007/s13277-014-2745-8
62. Jung J, Kim LJY, Wang X, Wu Q, Sanvoranart T, Hubert CG, Prager BC, Wallace LC, Jin X, Mack SC, Rich JN (2017) Nicotinamide metabolism regulates glioblastoma stem cell maintenance. JCI insight 2 (10). doi:10.1172/jci.insight.90019
63. Zhu T, Xie P, Gao YF, Huang MS, Li X, Zhang W, Zhou HH, Liu ZQ (2018) Nucleolar and spindle-associated protein 1 is a tumor grade correlated prognosis marker for glioma patients. CNS neuroscience & therapeutics 24 (3):178-186. doi:10.1111/cns.12803
64. Joel M, Mughal AA, Grieg Z, Murrell W, Palmero S, Mikkelsen B, Fjerdingstad HB, Sandberg CJ, Behnan J, Glover JC, Langmoen IA, Stangeland B (2015) Targeting PBK/TOPK decreases growth and survival of glioma initiating cells in vitro and attenuates tumor growth in vivo. Molecular cancer 14:121. doi:10.1186/s12943-015-0398-x
65. Ma K, Cheng Z, Sun L, Li H (2017) Identification of potential therapeutic targets for gliomas by bioinformatics analysis. Oncology letters 14 (5):5203-5210. doi:10.3892/ol.2017.6850
66. Li Y, Wang W, Wang F, Wu Q, Li W, Zhong X, Tian K, Zeng T, Gao L, Liu Y, Li S, Jiang X, Du G, Zhou Y (2017) Paired related homeobox 1 transactivates dopamine D2 receptor to maintain propagation and tumorigenicity of glioma-initiating cells. Journal of molecular cell biology 9 (4):302-314. doi:10.1093/jmcb/mjx017
67. Yang BY, Song JW, Sun HZ, Xing JC, Yang ZH, Wei CY, Xu TY, Yu ZN, Zhang YN, Wang YF, Chang H, Xu ZP, Hou M, Ji MJ, Zhang YS (2018) PSMB8 regulates glioma cell migration, proliferation, and apoptosis through modulating ERK1/2 and PI3K/AKT signaling pathways. Biomedicine & pharmacotherapy = Biomedecine & pharmacotherapie 100:205-212. doi:10.1016/j.biopha.2018.01.170
68. Tung JN, Ko CP, Yang SF, Cheng CW, Chen PN, Chang CY, Lin CL, Yang TF, Hsieh YH, Chen KC (2016) Inhibition of pentraxin 3 in glioma cells impairs proliferation and invasion in vitro and in vivo. Journal of neuro-oncology 129 (2):201-209. doi:10.1007/s11060-016-2168-z
69. Liu Y, Carson-Walter EB, Cooper A, Winans BN, Johnson MD, Walter KA (2010) Vascular gene expression patterns are conserved in primary and metastatic brain tumors. Journal of neuro-oncology 99 (1):13-24. doi:10.1007/s11060-009-0105-0
70. Abbadi S, Rodarte JJ, Abutaleb A, Lavell E, Smith CL, Ruff W, Schiller J, Olivi A, Levchenko A, Guerrero-Cazares H, Quinones-Hinojosa A (2014) Glucose-6-phosphatase is a key metabolic regulator of glioblastoma invasion. Molecular cancer research : MCR 12 (11):1547-1559. doi:10.1158/1541-7786.MCR-14-0106-T
71. Ciriello G, Cerami E, Sander C, Schultz N (2012) Mutual exclusivity analysis identifies oncogenic network modules. Genome research 22 (2):398-406. doi:10.1101/gr.125567.111
72. Ferrarese R, Harsh GRt, Yadav AK, Bug E, Maticzka D, Reichardt W, Dombrowski SM, Miller TE, Masilamani AP, Dai F, Kim H, Hadler M, Scholtens DM, Yu IL, Beck J, Srinivasasainagendra V, Costa F, Baxan N, Pfeifer D, von Elverfeldt D, Backofen R, Weyerbrock A, Duarte CW, He X, Prinz M, Chandler JP,

Vogel H, Chakravarti A, Rich JN, Carro MS, Bredel M (2014) Lineage-specific splicing of a brain-enriched alternative exon promotes glioblastoma progression. The Journal of clinical investigation 124 (7):2861-2876. doi:10.1172/JCI68836

73. Weiler M, Pfenning PN, Thiepold AL, Blaes J, Jestaedt L, Gronych J, Dittmann LM, Berger B, Jugold M, Kosch M, Combs SE, von Deimling A, Weller M, Bendszus M, Platten M, Wick W (2013) Suppression of proinvasive RGS4 by mTOR inhibition optimizes glioma treatment. Oncogene 32 (9):1099-1109. doi:10.1038/onc.2012.137
74. Liu Y, Wang F, Liu Y, Yao Y, Lv X, Dong B, Li J, Ren S, Yao Y, Xu Y (2016) RNF135, RING finger protein, promotes the proliferation of human glioblastoma cells in vivo and in vitro via the ERK pathway. Scientific reports 6:20642. doi:10.1038/srep20642
75. Newman WC, Kim WJ, Amankulor NM (2017) BRCA1-Regulated RRM2 Expression Protects Glioblastoma Cells from Endogenous Replication Stress and Promotes Tumorigenicity. Neurosurgery 80 (5):N34. doi:10.1093/neuros/nyx106
76. Roy A, Coum A, Marinescu VD, Polajeva J, Smits A, Nelander S, Uhrbom L, Westermark B, Forsberg-Nilsson K, Ponten F, Tchougounova E (2015) Glioma-derived plasminogen activator inhibitor-1 (PAI-1) regulates the recruitment of LRP1 positive mast cells. Oncotarget 6 (27):23647-23661. doi:10.18632/oncotarget.4640
77. Dai J, Bing Z, Zhang Y, Li Q, Niu L, Liang W, Yuan G, Duan L, Yin H, Pan Y (2017) Integrated mRNAseq and microRNAseq data analysis for grade III gliomas. Molecular medicine reports 16 (5):7468-7478. doi:10.3892/mmr.2017.7545
78. Leone PE, Gonzalez MB, Elosua C, Gomez-Moreta JA, Lumbreras E, Robledo C, Santos-Briz A, Valero JM, de la Guardia RD, Gutierrez NC, Hernandez JM, Garcia JL (2012) Integration of global spectral karyotyping, CGH arrays, and expression arrays reveals important genes in the pathogenesis of glioblastoma multiforme. Annals of surgical oncology 19 (7):2367-2379. doi:10.1245/s10434-011-2202-5
79. Jiang L, Zhou J, Zhong D, Zhou Y, Zhang W, Wu W, Zhao Z, Wang W, Xu W, He L, Ma Y, Hu Y, Zhang W, Li J (2017) Overexpression of SMC4 activates TGFbeta/Smad signaling and promotes aggressive phenotype in glioma cells. Oncogenesis 6 (3):e301. doi:10.1038/oncsis.2017.8
80. Chang KY, Hsu TI, Hsu CC, Tsai SY, Liu JJ, Chou SW, Liu MS, Liou JP, Ko CY, Chen KY, Hung JJ, Chang WC, Chuang CK, Kao TJ, Chuang JY (2017) Specificity protein 1-modulated superoxide dismutase 2 enhances temozolomide resistance in glioblastoma, which is independent of O(6)-methylguanine-DNA methyltransferase. Redox biology 13:655-664. doi:10.1016/j.redox.2017.08.005
81. Korkolopoulou P, Levidou G, El-Habr EA, Adamopoulos C, Fragkou P, Boviatsis E, Themistocleous MS, Petraki K, Vrettakos G, Sakalidou M, Samaras V, Zisakis A, Saetta A, Chatziandreou I, Patsouris E, Piperi C (2013) Sox11 expression in astrocytic gliomas: correlation with nestin/c-Met/IDH1-R132H expression phenotypes, p-Stat-3 and survival. British journal of cancer 108 (10):2142-2152. doi:10.1038/bjc.2013.176
82. Zhang J, Jiang H, Shao J, Mao R, Liu J, Ma Y, Fang X, Zhao N, Zheng S, Lin B (2014) SOX4 inhibits GBM cell growth and induces G0/G1 cell cycle arrest through Akt-p53 axis. BMC neurology 14:207. doi:10.1186/s12883-014-0207-y
83. Yuan ZS, Cao Y, Li ZY (2017) IGFBP2 induces SPRY1 expression via NF-kappaB signaling pathway in glioblastoma multiforme (GBM). European review for medical and pharmacological sciences 21 (22):5072-5080. doi:10.26355/eurrev_201711_13821
84. Wei B, Wang L, Du C, Hu G, Wang L, Jin Y, Kong D (2015) Identification of differentially expressed genes regulated by transcription factors in glioblastomas by bioinformatics analysis. Molecular medicine reports 11 (4):2548-2554. doi:10.3892/mmr.2014.3094
85. Liu Y, Hu H, Wang K, Zhang C, Wang Y, Yao K, Yang P, Han L, Kang C, Zhang W, Jiang T (2014) Multidimensional analysis of gene expression reveals TGFB1I1-induced EMT contributes to malignant progression of astrocytomas. Oncotarget 5 (24):12593-12606. doi:10.18632/oncotarget.2518

86. Pan YB, Zhang CH, Wang SQ, Ai PH, Chen K, Zhu L, Sun ZL, Feng DF (2018) Transforming growth factor beta induced (TGFBI) is a potential signature gene for mesenchymal subtype high-grade glioma. Journal of neuro-oncology 137 (2):395-407. doi:10.1007/s11060-017-2729-9
87. Groft LL, Muzik H, Rewcastle NB, Johnston RN, Knauper V, Lafleur MA, Forsyth PA, Edwards DR (2001) Differential expression and localization of TIMP-1 and TIMP-4 in human gliomas. British journal of cancer 85 (1):55-63. doi:10.1054/bjoc.2001.1854
88. Binder ZA, Siu IM, Eberhart CG, Ap Rhys C, Bai RY, Staedtke V, Zhang H, Smoll NR, Piantadosi S, Piccirillo SG, Dimeco F, Weingart JD, Vescovi A, Olivi A, Riggins GJ, Gallia GL (2013) Podocalyxin-like protein is expressed in glioblastoma multiforme stem-like cells and is associated with poor outcome. PloS one 8 (10):e75945. doi:10.1371/journal.pone.0075945
89. Sun W, Qiu G, Zou Y, Cai Z, Wang P, Lin X, Huang J, Jiang L, Ding X, Hu G (2015) Knockdown of TMEM45A inhibits the proliferation, migration and invasion of glioma cells. International journal of clinical and experimental pathology 8 (10):12657-12667
90. Lan J, Huang HY, Lee SW, Chen TJ, Tai HC, Hsu HP, Chang KY, Li CF (2014) TOP2A overexpression as a poor prognostic factor in patients with nasopharyngeal carcinoma. Tumour biology : the journal of the International Society for Oncodevelopmental Biology and Medicine 35 (1):179-187. doi:10.1007/s13277-013-1022-6
91. Gu JJ, Zhang JH, Chen HJ, Wang SS (2016) TPX2 promotes glioma cell proliferation and invasion via activation of the AKT signaling pathway. Oncology letters 12 (6):5015-5022. doi:10.3892/ol.2016.5371
92. Mahadev V, Starr R, Wright SL, Martinez C, Jensen MC, Barish ME, Forman SJ, Brown CE (2014) Cytokine induction of VCAM-1 but not IL13Ralpha2 on glioma cells: a tale of two antibodies. PloS one 9 (5):e95123. doi:10.1371/journal.pone.0095123
93. Zhang XH, Qian Y, Li Z, Zhang NN, Xie YJ (2016) Let-7g-5p inhibits epithelial-mesenchymal transition consistent with reduction of glioma stem cell phenotypes by targeting VSIG4 in glioblastoma. Oncology reports 36 (5):2967-2975. doi:10.3892/or.2016.5098
94. Hu B, Wang Q, Wang YA, Hua S, Sauve CG, Ong D, Lan ZD, Chang Q, Ho YW, Monasterio MM, Lu X, Zhong Y, Zhang J, Deng P, Tan Z, Wang G, Liao WT, Corley LJ, Yan H, Zhang J, You Y, Liu N, Cai L, Finocchiaro G, Phillips JJ, Berger MS, Spring DJ, Hu J, Sulman EP, Fuller GN, Chin L, Verhaak RGW, DePinho RA (2016) Epigenetic Activation of WNT5A Drives Glioblastoma Stem Cell Differentiation and Invasive Growth. Cell 167 (5):1281-1295 e1218. doi:10.1016/j.cell.2016.10.039
95. Luo Q, Huang H, Deng Y, Huang H, Fu H, Luo K, Li C, Qin C, Wei Z, Li X (2015) [Lentivirus-mediated shRNA targeting ZNF217 suppresses cell growth, migration, and invasion of glioma cells in vitro]. Nan fang yi ke da xue xue bao = Journal of Southern Medical University 35 (7):1024-1027, 1033
96. Wang Y, Liu T, Yang N, Xu S, Li X, Wang D (2016) Hypoxia and macrophages promote glioblastoma invasion by the CCL4-CCR5 axis. Oncology reports 36 (6):3522-3528. doi:10.3892/or.2016.5171
97. Liang J, Piao Y, Henry V, Tiao N, de Groot JF (2015) Interferon-regulatory factor-1 (IRF1) regulates bevacizumab induced autophagy. Oncotarget 6 (31):31479-31492. doi:10.18632/oncotarget.5491
98. Gao X, Mi Y, Ma Y, Jin W (2014) LEF1 regulates glioblastoma cell proliferation, migration, invasion, and cancer stem-like cell self-renewal. Tumour biology : the journal of the International Society for Oncodevelopmental Biology and Medicine 35 (11):11505-11511. doi:10.1007/s13277-014-2466-z
99. Donaires FS, Godoy PR, Leandro GS, Puthier D, Sakamoto-Hojo ET (2017) E2F transcription factors associated with up-regulated genes in glioblastoma. Cancer biomarkers : section A of Disease markers 18 (2):199-208. doi:10.3233/CBM-161628
100. Edlinger M, Strohmaier S, Jonsson H, Bjorge T, Manjer J, Borena WT, Haggstrom C, Engeland A, Tretli S, Concin H, Nagel G, Selmer R, Johansen D, Stocks T, Hallmans G, Stattin P, Ulmer H (2012) Blood pressure and other metabolic syndrome factors and risk of brain tumour in the large population-based Me-Can cohort study. Journal of hypertension 30 (2):290-296. doi:10.1097/HJH.0b013e32834e9176

101. Zhang D, Li Y, Wang R, Li Y, Shi P, Kan Z, Pang X (2016) Inhibition of REST Suppresses Proliferation and Migration in Glioblastoma Cells. International journal of molecular sciences 17 (5). doi:10.3390/ijms17050664
102. Laddha SV, Nayak S, Paul D, Reddy R, Sharma C, Jha P, Hariharan M, Agrawal A, Chowdhury S, Sarkar C, Mukhopadhyay A (2013) Genome-wide analysis reveals downregulation of miR-379/miR-656 cluster in human cancers. Biology direct 8:10. doi:10.1186/1745-6150-8-10
103. Choe G, Horvath S, Cloughesy TF, Crosby K, Seligson D, Palotie A, Inge L, Smith BL, Sawyers CL, Mischel PS (2003) Analysis of the phosphatidylinositol 3'-kinase signaling pathway in glioblastoma patients in vivo. Cancer research 63 (11):2742-2746
104. Chen ZH, Hu HK, Zhang CR, Lu CY, Bao Y, Cai Z, Zou YX, Hu GH, Jiang L (2016) Down-regulation of long non-coding RNA FOXD3 antisense RNA 1 (FOXD3-AS1) inhibits cell proliferation, migration, and invasion in malignant glioma cells. American journal of translational research 8 (10):4106-4119
105. Song H, Zhang Y, Liu N, Wan C, Zhang D, Zhao S, Kong Y, Yuan L (2016) miR-92b regulates glioma cells proliferation, migration, invasion, and apoptosis via PTEN/Akt signaling pathway. Journal of physiology and biochemistry 72 (2):201-211. doi:10.1007/s13105-016-0470-z
106. Ben-Hamo R, Zilberberg A, Cohen H, Efroni S (2016) hsa-miR-9 controls the mobility behavior of glioblastoma cells via regulation of MAPK14 signaling elements. Oncotarget 7 (17):23170-23181. doi:10.18632/oncotarget.6687
107. Conti A, Romeo SG, Cama A, La Torre D, Barresi V, Pezzino G, Tomasello C, Cardali S, Angileri FF, Polito F, Ferlazzo G, Di Giorgio R, Germano A, Aguennouz M (2016) MiRNA expression profiling in human gliomas: upregulated miR-363 increases cell survival and proliferation. Tumour biology : the journal of the International Society for Oncodevelopmental Biology and Medicine 37 (10):14035-14048. doi:10.1007/s13277-016-5273-x
108. Lavon I, Zrihan D, Granit A, Einstein O, Fainstein N, Cohen MA, Cohen MA, Zelikovitch B, Shoshan Y, Spektor S, Reubinoff BE, Felig Y, Gerlitz O, Ben-Hur T, Smith Y, Siegal T (2010) Gliomas display a microRNA expression profile reminiscent of neural precursor cells. Neuro-oncology 12 (5):422-433. doi:10.1093/neuonc/nop061
109. Xia Z, Qiu D, Deng J, Jiao X, Yang R, Sun Z, Wan X, Li J (2018) Methylation-induced downregulation and tumor-suppressive role of microRNA-98 in glioma through targeting Sal-like protein 4. International journal of molecular medicine 41 (5):2651-2659. doi:10.3892/ijmm.2018.3464
110. Chen L, Zhang W, Yan W, Han L, Zhang K, Shi Z, Zhang J, Wang Y, Li Y, Yu S, Pu P, Jiang C, Jiang T, Kang C (2012) The putative tumor suppressor miR-524-5p directly targets Jagged-1 and Hes-1 in glioma. Carcinogenesis 33 (11):2276-2282. doi:10.1093/carcin/bgs261
111. Tang H, Liu X, Wang Z, She X, Zeng X, Deng M, Liao Q, Guo X, Wang R, Li X, Zeng F, Wu M, Li G (2011) Interaction of hsa-miR-381 and glioma suppressor LRRC4 is involved in glioma growth. Brain research 1390:21-32. doi:10.1016/j.brainres.2011.03.034
112. Zhang A, Hao J, Wang K, Huang Q, Yu K, Kang C, Wang G, Jia Z, Han L, Pu P (2013) Down-regulation of miR-106b suppresses the growth of human glioma cells. Journal of neuro-oncology 112 (2):179-189. doi:10.1007/s11060-013-1061-2
113. Wang Z, Wang B, Shi Y, Xu C, Xiao HL, Ma LN, Xu SL, Yang L, Wang QL, Dang WQ, Cui W, Yu SC, Ping YF, Cui YH, Kung HF, Qian C, Zhang X, Bian XW (2015) Oncogenic miR-20a and miR-106a enhance the invasiveness of human glioma stem cells by directly targeting TIMP-2. Oncogene 34 (11):1407-1419. doi:10.1038/onc.2014.75
114. Xu G, Li JY (2016) Differential expression of PDGFRB and EGFR in microvascular proliferation in glioblastoma. Tumour biology : the journal of the International Society for Oncodevelopmental Biology and Medicine 37 (8):10577-10586. doi:10.1007/s13277-016-4968-3
115. Xi Z, Wang P, Xue Y, Shang C, Liu X, Ma J, Li Z, Li Z, Bao M, Liu Y (2017) Overexpression of miR-29a

reduces the oncogenic properties of glioblastoma stem cells by downregulating Quaking gene isoform 6. Oncotarget 8 (15):24949-24963. doi:10.18632/oncotarget.15327

116. Chen H, Li X, Li W, Zheng H (2015) miR-130a can predict response to temozolomide in patients with glioblastoma multiforme, independently of O6-methylguanine-DNA methyltransferase. Journal of translational medicine 13:69. doi:10.1186/s12967-015-0435-y

117. Hong L, Ya-Wei L, Hai W, Qiang Z, Jun-Jie L, Huang A, Song-Tao Q, Yun-Tao L (2016) MiR-519a functions as a tumor suppressor in glioma by targeting the oncogenic STAT3 pathway. Journal of neuro-oncology 128 (1):35-45. doi:10.1007/s11060-016-2095-z

118. Wang H, Sun T, Hu J, Zhang R, Rao Y, Wang S, Chen R, McLendon RE, Friedman AH, Keir ST, Bigner DD, Li QJ, Wang H, Wang XF (2014) miR-33a promotes glioma-initiating cell self-renewal via PKA and NOTCH pathways. The Journal of clinical investigation 124 (10):4489-4502. doi:10.1172/JCI75284

119. Liu X, Wang F, Tian L, Wang T, Zhang W, Li B, Bai YA (2016) MicroRNA-520b affects the proliferation of human glioblastoma cells by directly targeting cyclin D1. Tumour biology : the journal of the International Society for Oncodevelopmental Biology and Medicine 37 (6):7921-7928. doi:10.1007/s13277-015-4666-6
